# Novel GS5 sericin mitigates UVA-induced photoaging by activating Nrf2 and inhibiting the JAK-STAT pathway

**DOI:** 10.7150/ijbs.123702

**Published:** 2026-01-14

**Authors:** Haiqiong Guo, Yueting Sun, Wenyu Shi, Rui Huang, Qingxiu He, Ping Zhao, Qingyou Xia

**Affiliations:** 1 Biological Science Research Center, Integrative Science Center of Germplasm Creation in Western China (CHONGQING) Science City, Southwest University, Chongqing 400715, China.; 2 Key Laboratory for Germplasm Creation in Upper Reaches of the Yangtze River, Ministry of Agriculture and Rural Affairs, Chongqing 400715, China.

**Keywords:** ultraviolet A, Nrf2, PPI inhibitors, transgenic silkworms, GS5 sericin, anti-photoaging

## Abstract

Chronic ultraviolet (UV) exposure drives skin degeneration, causing photoaging and increased carcinogenesis risk. To address complex pathogenesis and limited treatments, we developed GS5, a novel anti-photoaging sericin. GS5 fuses natural sericin with Seq10, a Kelch-like ECH-associated protein 1 (Keap1)-nuclear factor erythroid 2-related factor 2 (Nrf2) protein-protein interaction (PPI) inhibitor identified through molecular docking/dynamics. Seq10 binds Keap1, activates Nrf2 transcription, and alleviates UVA-induced photoaging Nrf2-dependently. Given the application bottlenecks of peptide molecules, we efficiently expressed the GS5 recombinant protein using the silk gland reactor of the silkworm and optimized the enzymatic extraction process to obtain high-activity GS5 sericin. *In vitro*, GS5 outperformed wild-type (WT) sericin and Seq10, enhancing viability/proliferation in irradiated keratinocytes and fibroblasts while reducing senescence markers (Senescence-associated β-galactosidase (SA-β-gal), P21), reactive oxygen species (ROS), DNA damage, and inflammation. GS5's photoprotection mechanistically requires Nrf2 activation. *In vivo*, GS5 reversed skin damage in UVA-irradiated mice, improving appearance and histology. RNA-seq implicated Janus kinase (JAK)-signal transducer and activator of transcription (STAT) pathway inhibition via immune/inflammation-related gene modulation. This study innovatively combines a targeted PPI inhibitor with sericin to create GS5, which mitigates photoaging through dual Nrf2 activation and JAK-STAT inhibition, offering a safe, effective, and sustainable therapeutic strategy.

## 1. Introduction

The skin, the body's largest organ, provides the primary defense through microbial, physical, chemical, and innate immune barriers 1. Skin aging results from endogenous (intrinsic) and exogenous (environmental) factors that collectively alter the structure and function of epidermal, dermal, and subcutaneous layers 2, 3. Solar radiation constitutes the predominant exogenous accelerator, responsible for > 80% of facial aging. While ultraviolet (UV) radiation, particularly UVA (315-400 nm) which penetrates deeply into the dermis, is a principal driver of photoaging 4, emerging evidence highlights the significant role of high-energy visible (HEV) light, especially blue light (400-500 nm) 5. Both UVA and HEV light can penetrate the skin, generating reactive oxygen species (ROS) and inducing oxidative damage, inflammation, and extracellular matrix degradation through shared molecular pathways, making comprehensive photoprotection a critical yet unmet need 6, 7. Chronic UV exposure stimulates reactive oxygen species (ROS) overproduction, inducing cellular damage and senescence 8, 9. Excessive ROS activates the mitogen-activated protein kinase (MAPK)/activator protein-1 (AP-1) pathway, upregulating matrix metalloproteinases (MMPs) expression to degrade collagen while triggering the nuclear factor kappa-B (NF-κB) signaling 10, 11. These pathways collectively induce the senescence-associated secretory phenotype (SASP), characterized by MMPs and inflammatory mediators 12. Prolonged solar exposure also suppresses cutaneous immunity, compromising defense against pathogens and malignant cells, thereby increasing susceptibility to dermatological disorders including basal cell carcinoma 13.

Multiple photoaging treatments exist, including retinoids, sunscreens, antioxidants, and phototherapy 14, 15. However, many cause adverse effects (e.g., allergic reactions, irritation) or face biosafety, environmental, and photostability challenges 16, 17. While retinoids remain the gold standard for photoaging prevention, they induce cutaneous toxicity including xerosis, desquamation, and erythema 18. The limited efficacy and side effects of current therapies underscore the need for strategies that precisely target the fundamental molecular drivers of photoaging. Among these, oxidative stress triggered by solar radiation is a central pathological event, leading to inflammation, extracellular matrix (ECM) degradation, and cellular senescence 12. Consequently, enhancing the skin's endogenous antioxidant defense system represents a rational and powerful therapeutic approach. The transcription factor nuclear factor erythroid 2-related factor 2 (Nrf2) is the master regulator of cellular antioxidant response 19, 20. Under basal conditions, Nrf2 is constitutively inhibited by its cytoplasmic repressor, Kelch-like ECH-associated protein 1 (Keap1). Under oxidative stress, Nrf2 translocates to the nucleus and induces cytoprotective genes (e.g., reduced nicotinamide adenine dinucleotide phosphate (NAD(P)H) quinone oxidoreductase 1 (NQO1), heme oxygenase 1 (HO-1), and Glutamate-Cysteine Ligase Modifier Subunit (GCLM)) 21, 22. Therefore, disrupting the Keap1-Nrf2 protein-protein interaction (PPI) to activate Nrf2 offers a molecular strategy for preventing oxidative skin damage by bolstering the skin's intrinsic resilience.

Current small-molecule Keap1-Nrf2 inhibitors (e.g., methyl bardoxolone, sulforaphane) effectively activate antioxidant responses but face clinical limitations due to off-target effects and dose-dependent toxicity 23, 24. Peptide inhibitors, due to their highly defined structures and specific amino acid sequences, can precisely target the PPI interface, thereby reducing the risk of off-target effects. Their favorable biocompatibility and low toxicity profile position them as promising alternatives to conventional inhibitors 25, 26. Virtual screening of the Keap1-Nrf2 interaction interface (notably the Nrf2 Glu-Thr-Gly-Glu motif) identified competitive short peptide inhibitors that activate Nrf2 by disrupting Keap1 anchoring. However, translational applications in anti-photoaging remain constrained by poor transdermal delivery efficiency and short plasma half-lives 27.

Sericin, a natural silk glycoprotein rich in hydrophilic groups, shows promise for anti-photoaging research due to its unique structural and functional properties. This biocompatible protein, extracted from silk fibroin coatings, exhibits exceptional water solubility, moisturizing capacity, and antioxidant activity 28, 29. Studies confirm sericin scavenges free radicals, mitigates oxidative stress, and regulates apoptosis and inflammation, highlighting its therapeutic potential for skin repair and anti-aging applications 30, 31. Furthermore, its intrinsic porous structure and adsorptive properties enable utility as a drug delivery vehicle for targeted and sustained release of functional molecules 32.

In this study, targeting the Keap1 active pocket, we identified Seq10—a peptide inhibitor disrupting Keap1-Nrf2 PPI that activates Nrf2 and mitigates UVA-induced photoaging via Nrf2-dependent mechanisms. Given the shared oxidative mechanisms between UVA and HEV light, our findings may also provide a strategic basis for defending against broader solar radiation, including visible light. To overcome the inherent limitations of peptide transformation applications, we combined Seq10 with sericin to develop an enhanced anti-photoaging sericin, GS5. After optimizing the preparation process, GS5 demonstrated photoprotective efficacy in keratinocytes, fibroblasts, and UVA-photoaged mice. Mechanistic studies confirmed Nrf2-dependent protection, with RNA-seq revealing GS5-mediated rescue of UVA-induced photoaging via Janus kinase (JAK)-signal transducer and activator of transcription (STAT) pathway inhibition. Collectively, GS5 represents a novel anti-photoaging sericin modulating dual pathways, offering a promising strategy for photoaging intervention with significant translational potential.

## 2. Materials and methods

### 2.1 Molecular docking

Molecular docking was performed using the Surflex-Dock module in SYBYL-X 2.0. The crystal structure of Keap1 (PDB ID: 5WFV) served as the docking target. A peptide library was designed around the core Nrf2 motif Glu-Thr-Gly-Glu, targeting key Keap1 binding residues. Based on structure-activity relationship (SAR) studies 33, 34. Amino acids complementary to the Keap1 pocket were selected for variable positions (Y_1_/X_1_/X_2_/Y_2_) in the scaffold Ac-Y_1_-Asp-X_1_-Glu-Thr-Gly-Glu-X_2_-Y_2_-NH_2_. This generated a combinatorial library of 2250 peptides. Following energy minimization (Tripos force field), peptides were rapidly docked into the Keap1 active site (Tyr334, Ser363, Arg380, Asn414, Phe478, Arg483, Ser508, Tyr525, Gly530, Tyr572, Asp573, Phe577, and Ser602). The top 100 scoring hits underwent refined docking, with scores expressed as -log₁₀Kd 35. Resulting peptide-Keap1 complexes provided initial structures for subsequent all-atom molecular dynamics simulations.

### 2.2 Molecular dynamics simulations

Molecular dynamics (MD) simulations were performed using AMBER 18. Following docking, the top 14 ranked peptides underwent 200 ns MD simulations. The peptides and protein were described using the GAFF and ff14SB force fields, respectively. The complex was solvated in a TIP3P water box with a 10 Å buffer between the protein and the box edges, and neutralized with Na^+^/Cl^-^ ions to simulate physiological conditions. Simulations were performed using PMEMD 36, 37. Each simulation run lasted for 200 ns, with trajectories saved every 100 ps. Binding free energies were calculated via the Molecular Mechanics/Generalized Born Surface Area (MM/GBSA) method 38. Complex stability was assessed by monitoring the root mean square deviation (RMSD) over the final 100 ns of simulation.

### 2.3 Cell culture

HEK293T and human immortalized keratinocytes (HaCaTs) were obtained from Bina Biotechnology (Henan, China). Primary human dermal fibroblasts (HDFs) were sourced from Boxi Biotechnology (Guangdong, China). HaCaTs were maintained in RPMI 1640 medium (C11875500BT, Gibco, Thermo Fisher, Waltham, MA, USA), while HEK293T cells and HDFs were cultured in Dulbecco's modified Eagle medium (DMEM, C11965500BT, Gibco, Thermo Fisher, Waltham, MA, USA). All media were supplemented with 10% fetal bovine serum (FBS) and 1% streptomycin/penicillin (C11875500BT, Gibco, Thermo Fisher, Waltham, MA, USA). Cells were incubated at 37 °C with 5% CO₂ humidity and passaged using trypsin-EDTA.

### 2.4 Microscale thermophoresis (MST) assay

Protein-peptide binding affinities were quantified by MST (Monolith NT.115, NanoTemper Technologies, Munich, Germany). His-tagged Keap1 (50 nM) was fluorescently labeled (His-Tag Labeling Kit, MO-L018, NanoTemper Technologies, Munich, Germany) and titrated against serially diluted peptides (500 μM start, 16× two-fold dilutions). Capillary-loaded samples were analyzed at 40% light-emitting diode) LED/laser intensity using NT Analysis software (v1.4.23).

### 2.5 Nrf2-antioxidant response element (ARE) luciferase reporter assay

Nrf2 transcriptional activity was measured by dual-luciferase reporter assay. Cells co-transfected with Nrf2-promoter firefly luciferase and renilla control vectors (carring ARE gene) were peptide-treated 8 h post-transfection. After 24 h, luciferase activity was quantified (E1980, Promega, Madison, WI, USA) using t-BHQ (HY-100489, MCE, Merced, CA, USA) as positive control. Firefly signal was read after 10 min substrate incubation (Biotek Synergy H4, Vermont, USA), followed by renilla measurement post-Stop & Glo® addition. Nrf2 activity was calculated as normalized firefly/renilla ratio. Peptides were synthesized by GenScript (Nanjing, Jiangsu, China) (Table S1).

### 2.6 Cellular Thermal Shift Assay (CETSA)

CETSA measured target engagement via ligand-induced thermal stability shifts 39. HEK293T cells exposed to 50 µM Seq10 or (DMSO) (vehicle) for 12 h were lysed after temperature gradient treatment (3 min/point). After 3 min room-temperature equilibration and three liquid nitrogen freeze-thaw cycles, lysates were centrifuged (12,000 rpm, 15 min, 4 °C). Keap1 in supernatants was analyzed by western blot.

### 2.7 Cell viability assay

The proliferative effects of peptide Seq10, WT, and GS5 sericin on HaCaTs and HDFs were evaluated using the Cell Counting Kit-8 (CCK-8; C0042, Beyotime, Shanghai, China). Cells were seeded in 96-well plates (5×10³ cells/well) and treated with graded concentrations of each sample for 24 h. After adding 10 µl CCK-8 solution plus 90 µl medium per well, plates were incubated at 37 °C for 1 h. Absorbance was measured at 450 nm.

### 2.8 Cell photoaging model

HaCaTs and HDFs were treated with graded concentrations of samples for 24 h. After replacing medium with PBS, cells were irradiated with UVA (10 J/cm²) (HOPE Tianjin, China) on two consecutive days. Cells were then cultured for 12 or 24 h in sample-containing medium before photodamage assessment. Untreated/unirradiated cells served as controls.

### 2.9 Construction of a transgenic expression vector

To enhance Seq10 expression efficiency in silkworm, we generated a synthetic gene containing ten tandem repeats of its amino acid sequence to increase gene length. The coding sequence was codon-optimized for Bombyx mori and commercially synthesized (Genscript, Nanjing, China). Using BamHI/NotI restriction sites, this sequence was cloned into shuttle vector psL1180[hr3CQ-Ser1sp-DsRed-Ser1PA] to construct psL1180[hr3CQ-Ser1p-GS5-Ser1PA] 40. The expression cassette was then excised with AscI and inserted into the AscI site of pBac[3xp3DsRedSv40] vector 41, generating the final transgenic vector pBac[3×p3-DsRed-Sv40, hr3CQ-Ser1p-GS5-Ser1PA].

### 2.10 Generation of transgenic silkworms

We extracted ultra-pure plasmids of the piggyBac transgenic vector and helper vector 42 using the QIAGEN UltraPure Plasmid Extraction Kit (Cat. No. 12965, Qiagen, Hilden, Germany). After quantifying the plasmids at ~500 ng/µL, we mixed them at a 1:1 molar ratio and microinjected the solution into early embryos of the non-diapause silkworm strain D9L using an insect embryo microinjection syringe (IEMJ, Chongqing, China). Injected embryos were carefully incubated at 25 °C with 85% relative humidity. G0 larvae were reared to adulthood on mulberry leaves and outcrossed with wild-type moths to produce G1 eggs. At day 6-7, G1 eggs showing eye-specific red fluorescence under stereomicroscopy (Olympus, Tokyo, Japan) were selected. Moth-stage eye fluorescence confirmation yielded stable GS5-transgenic silkworm lines.

### 2.11 Expression analysis of GS5 recombinant protein in GS5 silk

GS5 silkworms were reared to cocooning. Cocoon pieces (GS5/WT) were dissolved in buffer (20 mM Tris-Cl, 8 M urea, pH 8.0) at 30 mg/mL, incubated at 80 °C for 30 min, and centrifuged (12,000 rpm, 15 min, 4 °C). Supernatants underwent SDS-PAGE with Coomassie R250 staining. Target bands (WT and GS5-specific) were excised, pre-treatment for mass spectrometry (MS) desalted, freeze-dried, reconstituted in 50 μL 0.1% formic acid, vortexed (30 s), and centrifuged (12,000 g, 10 min, room temperature). Samples were analyzed by MS (Qiantang Technology, Hangzhou, Zhejiang, China). Molecular weight discrepancies suggested glycosylation, prompting glycosylation-specific MS analysis.

### 2.12 Extraction of anti-photoaging GS5 sericin

Enzymatic hydrolysis enables efficient sericin degumming under mild conditions via protease-specific cleavage 43. As the amino acid sequence of Seq10 terminates in Tyr (Y) and lacks other cleavage sites, it is exclusively released as a single peptide by chymotrypsin (C8660, Solarbio, Beijing, China) digestion. Therefore, sericin degumming was achieved via chymotrypsin-mediated cleavage. Cocoon powder (1:30 w/v in ultrapure water) was sterilized (85 °C, 15 min), cooled, and optimization for extraction. Post-hydrolysis, enzymes were inactivated (85 °C, 15 min), samples centrifuged (12,000 rpm, 20 min), and supernatants collected. Hydrolysis degree (DH) was quantified by o-phthalaldehyde (OPA) assay 44. SDS-PAGE analyzed sericin extracts, with 10 kDa ultrafiltration separating high/low-MW fractions. Antioxidant activity was assessed via 2,2'-azino-bis (3-ethylbenzthiazoline-6-sulfonic acid) (ABTS) scavenging (S0119, Beyotime, Shanghai, China) and Ferric Reducing Ability of Plasma (FRAP) (S0116, Beyotime, Shanghai, China) assays following manufacturer protocols. Enzymatically sericin was lyophilized and stored at -80 °C.

### 2.13 Cell transfection

Cells were seeded in 6-well plates and transfected at 50-70% confluence using X-treme GENE™ siRNA (4476093001, Roche, Basel, Switzerland) according to manufacturer instructions. Human small interfering RNA (siRNA) oligomers (50 nM, Sangon Biotech, Shanghai, China) (Table S2) were diluted in Opti-MEM medium (31985070, Thermo Fisher, MA, USA). Fresh medium was added 8 h post-transfection, and subsequent experiments were performed after 24 h.

### 2.14 Western blotting

Total protein was extracted using RIPA lysis buffer (P0013B, Beyotime, Shanghai, China) containing protease inhibitors and quantified by BCA assay (ZJ101, Epizyme Biotech, Shanghai, China). Proteins (20 µg) were separated by 10% SDS-PAGE and transferred to a polyvinylidene fluoride (PVDF) membrane (IPFL00010, Millipore, USA). After blocking with a protein-free rapid blocking buffer (PS108, Epizyme Biotech, Shanghai, China) at room temperature for 10 min, membranes were probed with primary and secondary antibodies (Table S3). Following TBST washes, proteins were visualized using ultra-sensitive ECL chemiluminescent reagent (34096, Thermo Fisher, Massachusetts, USA) on an imaging system (ChampChemi, Beijing, China), with band density quantified via ImageJ.

### 2.15 Quantitative real-time polymerase chain reaction(qRT-PCR)

Total RNA was extracted using the Steady Pure Rapid RNA Extraction Kit (AG21023, Accurate Biology, Hunan, China) and reverse-transcribed to cDNA with NovoScript®Plus All-in-one 1st Strand cDNA Synthesis SuperMix (E047, Novoprotein, Shanghai, China). qPCR was performed on a qTOWER3 system (Analytik Jena, Jena, Germany) using NovoStart®SYBR qPCR SuperMix Plus with gene-specific primers. GAPDH served as the endogenous control. Gene expression was quantification using both the standard curve and 2^(-ΔΔCt) method. The primer sequences used in this study are listed in Table S4.

### 2.16 Immunofluorescence staining

Specifically, the following procedures were performed on the cells: fixation with 4% paraformaldehyde, permeabilization with 0.2% Triton X-100 (P0096, Beyotime, Shanghai, China) in PBS, blocking with 5% goat serum (C0265, Beyotime, Shanghai, China) for 30 min, and overnight incubation with primary antibody at 4 °C. After secondary antibody incubation for 1 h at room temperature, nuclei were counterstained with DAPI (P0131, Beyotime, Shanghai, China) for 5 min in the dark. Protein localization was analyzed using a laser scanning confocal microscope (Zeiss LSM 800 with airscan, Oberkochen, Baden-Wurttemberg, Germany).

### 2.17 ROS detection

Intracellular ROS levels were measured using the fluorescent probe DCFH-DA. (S0033S, Beyotime, Shanghai, China). Following drug treatment, cells were incubated with 10 μM DCFH-DA working solution (30 min, 37 °C). After three washes with serum-free medium to remove excess probe, nuclei were stained with Hoechst. Fluorescence images were acquired using an EVOS FL microscope (Life Technologies, Carlsbad, California, USA) and quantified with ImageJ.

### 2.18 5-Ethynyl-2'-deoxyuridine (EdU) staining

Cell proliferation was assessed by EdU assay (AF555, Beyotime, Shanghai, China). Cells (2×10⁴/well in 24-well plates) underwent drug/UVA treatment, then 2 h incubation with 37 °C EdU solution. After fixation (15 min, 25 °C) and permeabilization (15 min), samples were incubated with Click reaction solution (30 min, dark, 25 °C). Nuclei were counterstained with Hoechst 33342 and imaged by fluorescence microscopy.

### 2.19 Senescence β-Galactosidase (SA-β-gal) staining

After thorough PBS washing, cells were fixed in 4% paraformaldehyde for 15 min at room temperature. Following three PBS washes, cells were incubated overnight with β-galactosidase staining solution (C0602, Beyotime, Shanghai, China) at 37 °C. Senescence-positive cells were quantified using ImageJ based on microscopic examination of three random fields per well.

### 2.20 Migration assay

Cells were seeded in 6-well plates and grown to form a monolayer. Monolayers were scratch-wounded using 200 µL pipette tip, washed with PBS to remove debris, and imaged at 0, 12, and 24 h post-scratching. Wound width was measured at predetermined locations using ImageJ. Migration rate was calculated as: (Initial wound area-Remaining wound area) / Initial wound area×100%.

### 2.21 Transwell assay

Cells were serum-starved and suspended in 1% FBS medium (3×10⁵ cells/mL). A 100 µL aliquot was placed into the upper chamber of an 8-µm pore Transwell insert (FTW043, Beyotime, Shanghai, China). The lower chamber contained 500 µL of 20% FBS medium. After 24 h incubation, non-migrated cells on the upper membrane surface were removed with a cotton swab following PBS wash and fixation with 4% paraformaldehyde. Migrated cells on the underside were stained with 0.1% crystal violet.

### 2.22 Mouse photoaging model and treatments

Specific pathogen-free (SPF) Kunming mice (Enswell Biology, Chongqing, China) were housed at 25 ± 2 °C, 40-60% humidity, 12 h light/dark cycle with ad libitum access to water and food. Dorsally depilated mice received thrice-weekly 5 J/cm² UVA irradiation (HOPE-MED 8130C, Tianjin, China) for 8 weeks, and were randomly assigned to six groups (n = 6/group): (a) Control (PBS gel); (b) UVA irradiation (UVA + PBS gel); (c) UVA + 0.1% WT sericin gel; (d) UVA + 0.1% Seq10 peptide gel; (e) UVA + 0.1% GS5 sericin gel; (f) UVA + 0.05% all-trans retinoic acid (ATRA Cream U.S.P.). WT, GS5, and Seq10 were homogenized in 4% sodium carboxymethyl cellulose (CMC-Na) containing 10% glycerin to prepare 0.1% (w/v) gels. Test compounds (1 mg/cm²) or PBS vehicle were applied post-irradiation. Body weights, dorsal skin hydration, and melanin content were monitored. After 4-week treatment, mice were euthanized. Skin samples were fixed in 4% paraformaldehyde (pH 7.4, 24 h, 4 °C), dehydrated, paraffin-embedded, and sectioned at 4 μm. Histopathological analysis employed modified hematoxylin and eosin (H&E) (G1121, Solarbio, Beijing, China) and a modified Masson's trichrome staining (G1346, Solarbio, Beijing, China).

### 2.23 Immunohistochemistry (IHC) assay

Following antigen retrieval and endogenous peroxidase blockade, paraffin-embedded skin sections were blocked with goat serum and incubated overnight with primary antibody at 4 °C. Sections were then incubated for 1 h with HRP-conjugated secondary antibody (A0208, Beyotime, Shanghai, China) at room temperature. Immunoreactivity was visualized using DAB substrate (P0202, Beyotime, Shanghai, China), counterstained with hematoxylin, hydrochloric acid-differentiated, dehydrated and mounted. DAB-positive areas were quantified from microscopy images using ImageJ.

### 2.24 Quantification of oxidative stress biomarkers

Skin tissue homogenates were centrifuged at 12,000 rpm for 15 min at 4 °C, and supernatants collected for analysis. Activity of catalase (CAT) (S0051, Beyotime, Shanghai, China), superoxide dismutase (SOD) (S0101S, Beyotime, Shanghai, China), and levels of malondialdehyde (MDA) (S0131S, Beyotime, Shanghai, China) were measured using commercial kits. Interleukin (IL)-1β (PDEM100209, Elabscience, Wuhan, Hubei, China) and IL-6 (E-EL-M0044, Elabscience, Wuhan, Hubei, China) levels were quantified by enzyme linked immunosorbent assay (ELISA) kits per manufacturer's protocol.

### 2.25 RNA-seq analysis

Total RNA was extracted with TRIzol® (9109, Takara, Tokyo, Japan) from treated HaCaTs. RNA sequencing and analysis followed established methods (Qiantang Biology, Suzhou, Jiangsu, China). DESeq2 identified differentially expressed genes (DEGs) between groups, visualized via volcano plot (ggplot2) and heatmap. Functional enrichment analysis of DEGs was performed using Database for Annotation, Visualization, and Integrated Discovery (DAVID) and Kyoto Encyclopedia of Genes and Genomes (KEGG) Orthology Based Annotation System (KOBAS), including Gene Ontology (GO) functional and KEGG pathway annotation.

### 2.26 Statistics and reproducibility

Graphing and statistical analyses were performed using GraphPad Prism version 8.0. All data are presented as mean ± standard deviation (SD) from a minimum of three independent experiments. Statistical analyses were conducted using one- or two-way analysis of variance (ANOVA). *P* = 0.05 or less was deemed significant. ∗*P* ≤ 0.05, ∗∗*P* ≤ 0.01, ∗∗∗*P* ≤ 0.001, and ∗∗∗∗*P* ≤ 0.0001 respectively. Non-significant differences are indicated as n.s.

## 3. Results

### 3.1 Structure-based screening of inhibitors targeting Keap1-Nrf2 PPIs

**Figure 1:**
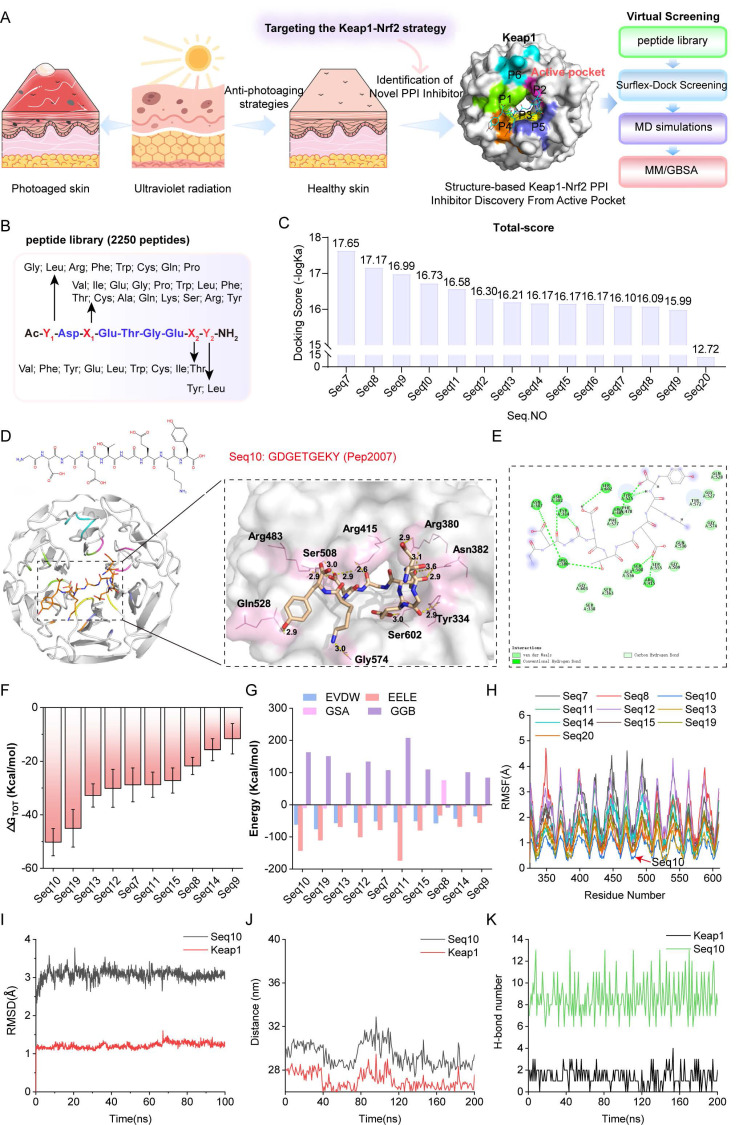
Screening of structure-based Keap1**-**Nrf2 PPI inhibitors. (A) The structure of Keap1 and the virtual screening model for discovering anti-photoaging inhibitors of the Keap1**-**Nrf2 PPI. (B) The virtual screening peptide library created using “Glu-Thr-Gly-Glu” as the template sequence. (C) Docking scores of candidate peptides selected based on molecular docking. (D) Schematic diagram of molecular docking indicating the binding mode between Seq10 and Keap1. (E) Two-dimensional schematic of the interaction between Seq10 and Keap1. (F) Assessment of the total binding energy of the peptide**-**Keap1 complex through molecular dynamics simulations. (G) Energy decomposition of the total binding energy of the peptide**-**Keap1 complex calculated using the MM/GBSA method. (H-K) The stability of the Seq10-Keap1 binding is evaluated through RMSF (H), RMSD (I), distance variation (J), and the number of hydrogen bonds (K).

The Keap1**-**Nrf2 binding interface contains a well-defined cavity (P1**-**P6). In this study, this cavity is designated as an active pocket, and a screening strategy was established to identify potential Keap1-Nrf2 PPI inhibitors (Fig. 1A). A peptide library containing 2250 peptides was constructed based on the “Glu-Thr-Gly-Glu” sequence template (Fig. 1B). Using the Surflex-Dock GeomX precision docking method, the top 14 potential peptides were selected (Fig. 1C). By analyzing the interaction patterns between Keap1 and the Glu-Thr-Gly-Glu motif in the crystal structure, we selected candidate peptides that primarily interact with the sub-pocket of the Keap1 active site, eliminating Seq20. Molecular docking revealed the spatial orientation and binding conformation of the candidate peptides with Keap1 (Fig. 1D and Fig. S1). The hydrogen bonds formed between the complexes enhance the stability of the binding. Additionally, two-dimensional interaction maps provide a detailed visualization of specific non-covalent interactions between the protein and ligand molecules. As shown in Fig. 1E, Seq10 binds to Keap1 primarily through van der Waals forces and hydrogen bonding. Fig. S2 also highlights hydrophobic interactions among other peptide complexes. Notably, Seq11, Seq17, Seq18, and Seq19 exhibit unfavorable interactions with Keap1. A 200 ns MD simulation further elucidates the molecular binding mechanism of the peptide-Keap1 interaction and evaluates the binding stability. During the MD process, Seq17, and Seq18 were eliminated due to their inability to form stable complexes, and no further research was conducted on them. The total binding free energy of the peptide-Keap1 complex was analyzed, with Fig. 1F showing that Seq10 exhibits the most favorable (negative) total binding energy compared to the other peptides. The MM/GBSA method for decomposing binding free energy indicates that electrostatic interactions and van der Waals forces contribute significantly to the total binding energy, with non-polar interactions (EVDW + GSA) playing a dominant role in peptide binding to Keap1 (Fig. 1G). Root means square fluctuation (RMSF) analysis reveals that Seq10 demonstrates more stable fluctuations during MD process (Fig. 1H). Furthermore, the stability of the interactions between the candidate peptides and Keap1 was evaluated through RMSD, distance changes, and hydrogen bond analysis. For candidate peptides, the RMSD values of Seq10 and Keap1 fluctuate within a range of 3.0 Å, indicating that the complex maintains greater stability (Fig. 1I and Fig. S3). The stability in terms of distance is particularly pronounced for Seq10, which varies within a range of 29 nm (Fig. 1J and Fig. S4). Regarding hydrogen bond interactions, the Seq10 complex system generates a greater number of hydrogen bonds during the MD process (Fig. 1K and Fig. S5), which is more conducive to binding stability.

**Figure 2:**
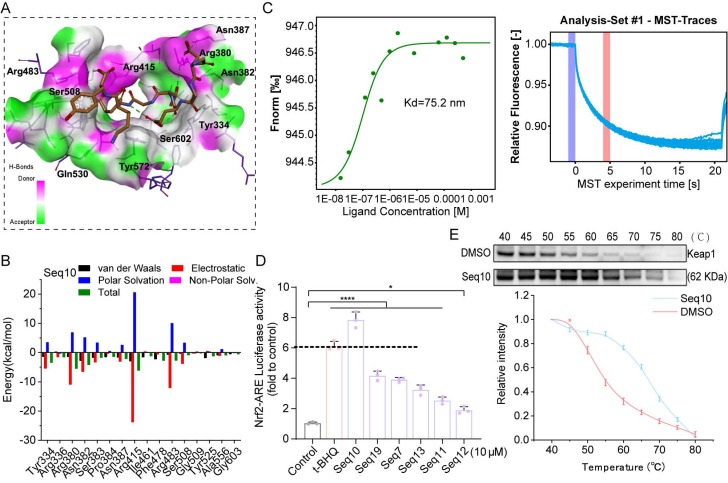
Identification of the binding ability between Seq10 and Keap1. (A) The binding mode of Seq10 and Keap1 after molecular dynamics simulation, with hydrogen bonds represented by dashed lines. (B) Contribution of different amino acid residues in the Seq10**-**Keap1 binding calculated using the MM/GBSA method. (C) Precise measurement of the binding affinity between Seq10 and Keap1 using MST. (D) ARE-luciferase reporter assay to determine whether Seq10 activates Nrf2-luciferase transcriptional activity. (E) CETSA to assess the thermal stability of Keap1 protein in cells treated with Seq10. All data are presented as means ± standard deviation (SD) (n = 3). Statistical significance is denoted by * P < 0.05, ** P < 0.01, *** P < 0.001, and ****P < 0.0001 vs. UVA-irradiated group.

Seq10 demonstrated superior binding potential among candidate peptides. Post-MD simulation analysis revealed a conserved binding mode featuring the “Glu-Thr-Gly-Glu” motif, with the peptide backbone occupying the P1-5 sub-pocket and extending into the active site (Fig. 2A). Hydrogen bonds (green dotted lines) stabilized the complex, exhibiting greater conformational stability than initial docking poses. MM/GBSA free energy decomposition identified electrostatic interactions as primary stabilizing forces, supplemented by van der Waals contributions, while polar solvation impeded binding (Fig. 2B). MST binding assays confirmed strong Seq10-Keap1 interaction (Kd = 75.2 nM; Fig. 2C). Seq10 also induced the highest Nrf2-ARE transcriptional activation, exceeding t-BHQ controls (Fig. 2D). Subsequently, CETSA analysis demonstrated direct binding through enhanced Keap1 thermal stability in HaCaTs versus DMSO (Fig. 2E). Collectively, these findings suggest that Seq10 binds Keap1 and activates Nrf2 transcriptional activity.

### 3.2 Seq10 mitigates UVA-induced cellular photoaging by activating Nrf2

**Figure 3:**
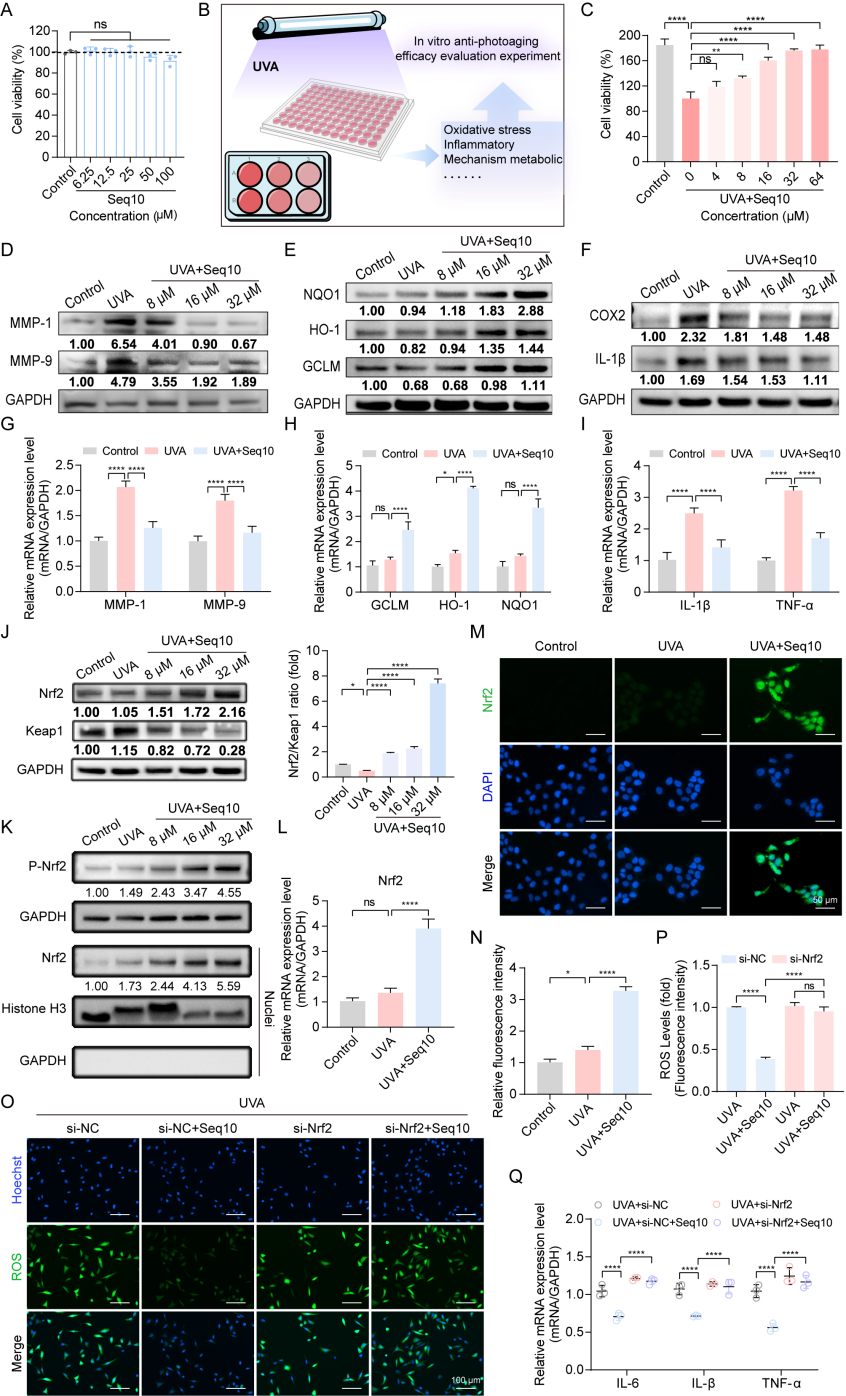
Seq10 demonstrates good potential for anti-photoaging and activation of the Nrf2 signaling pathway. Seq10 demonstrates good potential for photostability and activation of the Nrf2 signaling pathway. (A) HaCaTs were treated with specified concentrations of Seq10, and cell viability was assessed using the CCK8 assay. (B) A cellular photaging model was established to evaluate the photostability potential of Seq10. (C) The effect of Seq10 treatment on the viability of HaCaTs subjected to UVA irradiation. (D) The impact of Seq10 treatment on the expression levels of MMP-1 and MMP-9 proteins within cells. (E) The effect of Seq10 treatment on the expression levels of NQO1, HO-1, and GCLM proteins within cells. (F) The influence of Seq10 treatment on the expression levels of inflammatory factors COX2 and IL-1β proteins within cells. (G-I) Relative mRNA expression of MMP-1, MMP-9, GCLM, HO-1, NQO1, IL-1β, and TNF-α in each group. (J) The effect of Seq10 treatment on the protein levels of Nrf2 and Keap1 within cells. (K) The expression levels of P-Nrf2 in HaCaTs whole cell lysates and Nrf2 in the nucleus were processed in Seq10. (L) Quantification of Nrf2 released from HaCaTs by qRT-PCR. (M) Immunofluorescence staining of Nrf2 protein in HaCaTs. Scale bar, 50 µm. (N) Fluorescence intensity of ROS levels. (O) Analysis of intracellular ROS levels after Nrf2 knockdown. Scale bar, 100 µm. (P) Fluorescence intensity of ROS levels. (Q) Quantification of mRNA for IL-6, IL-1β, and TNF-α secreted by HaCaTs following Nrf2 knockdown. All data are presented as means ± standard deviation (SD) (n = 3). Statistical significance is denoted by * P < 0.05, ** P < 0.01, *** P < 0.001, and ****P < 0.0001 vs. UVA-irradiated group.

We first evaluated the cytotoxicity of Seq10 on HaCaTs, and we discovered that Seq10 showed no significant cytotoxicity even at 100 µM (Fig. 3A). We established UVA-induced cellular photoaging model to evaluate the *in vitro* anti-photoaging effects of Seq10 (Fig. 3B). In a UVA-induced photoaging model (10 J/cm²), Seq10 (4**-**64 µM) dose-dependently increased viability of irradiated HaCaTs (Fig. 3C). These doses were found to provide significant protection to HaCaTs against UVA damage and were used in subsequent experimental. UVA irradiation activates the secretion and expression of MMPs, which are markers of skin aging 45. We measured the secretion of MMP-1 and MMP-9 in HaCaTs under UVA irradiation with or without Seq10 using Western blot and qRT-PCR. The results indicated that Seq10 significantly reduced UVA-induced secretion of MMP-1 and MMP-9 (Figs. 3D, G). UVA radiation induces oxidative stress in the skin, leading to oxidative damage 46. Western blot and qRT-PCR analyses revealed that Seq10 treatment significantly upregulated the expression of NQO1, HO-1, and GCLM (Figs. 3E, H). Additionally, UVA irradiation caused a significant increase in inflammatory factors, which was effectively mitigated by Seq10 (Figs. 3F, I). Under basal conditions, Nrf2 activity is tightly regulated by its cytoplasmic interaction with Keap1. Upon external stimulation, Nrf2 becomes activated and translocates to the nucleus 47. Our findings showed that the Nrf2/Keap1 ratio increased in a dose-dependent manner in cells pre-treated with Seq10 (Fig. 3J). Furthermore, the significant increase in P-Nrf2 expression within the cells indicated that the treatment with Seq10 (32 µM) successfully activated the Nrf2 pathway. The detection of high levels of Nrf2 in the nuclear protein also signified the successful activation and nuclear translocation of Nrf2 (Fig. 3K). qRT-PCR analysis also corroborated that the mRNA expression level of Nrf2 in HaCaTs was significantly elevated following Seq10 treatment (Fig. 3L). Notably, immunofluorescence staining in HaCaTs revealed that Seq10 increased the amount of Nrf2 in the nucleus (Figs. 3M**-**N). NQO1, HO-1, and GCLM are downstream targets of Nrf2, and their increased expression levels alleviate cellular oxidative stress, further confirming Nrf2 activation. To investigate the role of Nrf2 in Seq10-mediated inhibition of cellular senescence, we conducted Nrf2 knockdown experiments in HaCaTs (Fig. S6). As shown in Figs. 3O**-**P, Nrf2 knockdown reversed the reduction in intracellular ROS generation, caused by Seq10. Furthermore, despite treatment with Seq10, Nrf2-deficient cells exhibited elevated levels of SASP (Fig. 3Q). These findings underscore the essential role of Nrf2 in the anti-photoaging effects of Seq10.

### 3.3 Production of GS5 anti-photoaging sericin in transgenic silkworms

**Figure 4:**
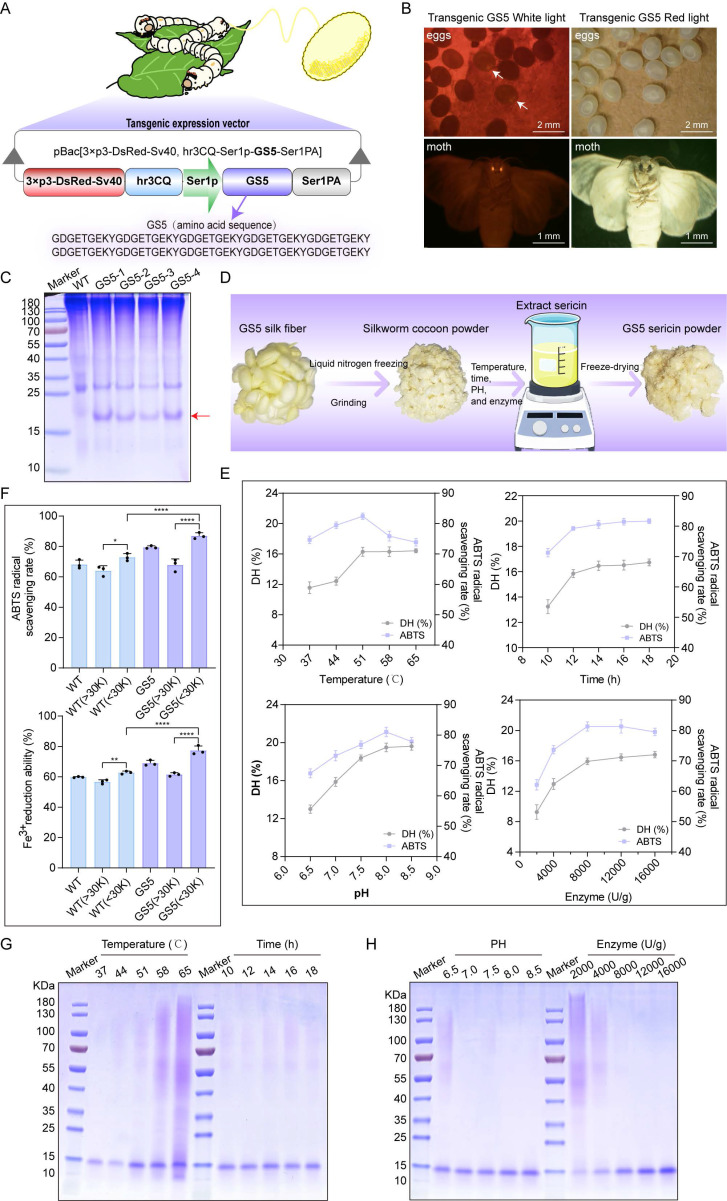
Production of GS5 anti-photoaging sericin protein from transgenic silkworms. (A) Schematic diagram of the transgenic GS5 expression vector. 3xp3DsRedSV40, Hr3, Ser1-P, and Ser1PA represent the transcription regulatory box of the DsRed fluorescent gene, the nuclear polyhedrosis virus enhancer, and the promoter and 3'-UTR of the sericin 1 gene. (B) Screening of positive transgenic silkworms in the G1 generation after microinjection by the presence of red fluorescent signals in the eyes. (C) SDS-PAGE analysis of GS5 recombinant protein from silkworm cocoon samples. The red arrow indicates the GS5 protein. (D) Schematic diagram of the extraction process of GS5 sericin powder. (E) Hydrolysis rates and activities of GS5 extracted under different temperature, time, pH, and enzyme quantity conditions. (F) Antioxidant activity of GS5 sericin protein solution with segmented molecular weight after ultrafiltration. (G-H) SDS-PAGE analysis of GS5 sericin protein solution extracted under different temperatures, times, pH, and enzyme quantity conditions. All data are presented as means ± standard deviation (SD) (n = 3). Statistical significance is denoted by * P < 0.05, ** P < 0.01, *** P < 0.001, and ****P < 0.0001 vs. UVA-irradiated group.

To find an environmentally friendly, highly bioactive, and biocompatible method for the sustainable production of Seq10, we turned our attention to a natural production platform―the silk gland bioreactor of the silkworm. Additionally, we noted the unique biological activity of sericin. The photoprotective biological activity of Seq10 can be maximized by fusing it with natural sericin. The Seq10 sequence was tandemly repeated tenfold (yielding GS5; 9.97 kDa theoretical MW) to enhance expression efficiency. GS5 was synthesized commercially and cloned into our established silk gland expression vector pBac[3×p3-DsRed-Sv40, hr3CQ-Ser1p-GS5-Ser1PA] (Fig. 4A). The transgenic vector was microinjected into 150 non-diapause silkworm eggs, resulting in 32 hatched embryos. After rearing to adulthood and interspecific hybridization, G1 progeny screening identified four transgenic lines with embryonic/adult eye-specific DsRed fluorescence (Fig. 4B), confirming successful piggyBac-mediated genomic integration. The successfully expressed GS5 gene did not compromise silkworm economic traits (Fig. S7). Through SDS-PAGE and LC-MS/MS analysis, the expression of the GS5 protein was successfully detected in transgenic silkworms (Fig. 4C, Figs. S8A**-**B). The observed molecular weight (19 kDa) exceeded theoretical predictions, prompting O-glycosylation prediction via online tools. Therefore, we conducted gel mass spectrometry (MS) analysis, which confirmed the prediction that O-glycosylation occurred at the position of threonine, resulting in an increase in molecular weight (Fig. S8C). Subsequently, we explored the optimal enzymatic hydrolysis conditions for chymotrypsin through a single-factor experiment, as illustrated in Fig. 4D. We optimized the reaction time, temperature, pH, and enzyme quantity sequentially to determine the optimal reaction conditions. The results indicated that the ideal enzymatic hydrolysis conditions were 51 °C, pH 8.0, 12 h incubation with 8000 U/g enzyme (Fig. 4E). Under these conditions, the highest degree of DH and ABTS radical scavenging rates were achieved, and a single band profile was observed on the SDS-PAGE gel, indicating complete enzymatic hydrolysis and purity of the products (Figs. 4G**-**H). Furthermore, the low molecular weight components obtained under the aforementioned conditions exhibited enhanced *in vitro* biological activity, suggesting that these components may represent the primary active forms and thus serve as the focus of subsequent research (Fig. 4F).

### 3.4 GS5 sericin alleviates photoaging of keratinocytes *in vitro*

HaCaTs mimic the characteristics of normal epidermal cells, and the epidermis, as the outermost layer of the skin, is directly exposed to UVA radiation 48. We first assessed the cytotoxicity of GS5 sericin on HaCaTs. Both WT and GS5 exhibited cytotoxicity-free profiles even at a concentration of 6.4 mg/mL and promoted cell proliferation at 0.4 mg/mL (Fig. S9A**-**B). To visualize cellular uptake, FITC-labeled GS5 was incubated with HaCaTs. Fluorescent signal appeared faintly at 2 h, and its intensity markedly increased from 4 h onward, demonstrating that GS5 was effectively internalized by HaCaTs (Fig. S10). To evaluate the protective effects of GS5 on HaCaTs, we investigated its effects on cell viability and proliferation after UVA irradiation. UVA exposure significantly decreased cell viability and proliferation, which were ameliorated by GS5 treatment.

**Figure 5:**
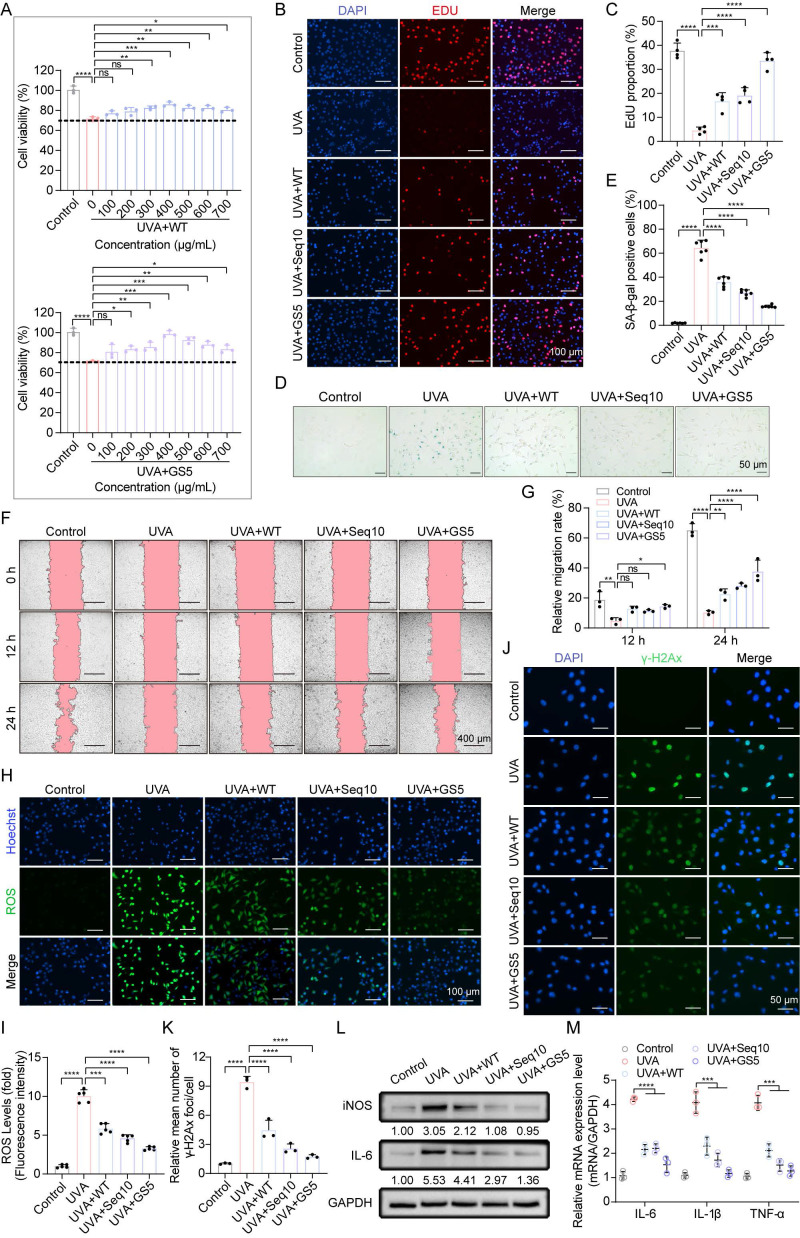
GS5 sericin protects keratinocytes from UVA-induced cellular senescence *in vitro*. (A) Effects of WT or GS5 sericin treatment on the viability of HaCaTs after UVA irradiation. (B) EdU (red) proliferation assay in HaCaTs. Hoechst (blue) staining for cell nuclei. Scale bar, 100 µm. (C) Proportion of EdU-labeled mitotic cells. (D) Representative images of SA-β-gal staining in HaCaTs. Scale bar, 50 μm. (E) Quantification of SA-β-gal positive cells. (F) Wound healing assay to determine the migratory capacity of HaCaTs. Scale bar, 400 μm. (G) Quantification of HaCaTs cell migration assay. (H) Representative immunofluorescence staining images of ROS (green) positive cells. Hoechst (blue) staining for cell nuclei. Scale bar, 100 μm. (I) Fluorescence intensity of ROS levels. (J) Representative immunofluorescence staining images of γ-H2Ax (green) positive cells. DAPI (blue) staining for cell nuclei. Scale bar, 50 μm. (K) Quantification of the average number of γ-H2Ax foci per cell. (L) Western blot analysis showing the change of IL-6 and iNOS in HaCaTs. (M) Quantification of IL-6, IL-1β, and TNF-α released by HaCaTs detected by qRT-PCR. All data are presented as means ± standard deviation (SD) (n = 3). Statistical significance is denoted by * P < 0.05, ** P < 0.01, *** P < 0.001, and ****P < 0.0001 vs. UVA-irradiated group.

Compared to WT and Seq10, GS5 demonstrated superior efficacy, achieving maximum recovery of cell viability at a concentration of 400 µg/mL (Figs. 5A**-**C). Based on these results, we selected 400 µg/mL for subsequent experiments. We then evaluated the effects of GS5 sericin on photoaging-associated biomarkers. UVA irradiation induced the accumulation of SA-β-gal positive cells in HaCaTs, which significantly reduced by GS5 sericin treatment (Figs. 5D**-**E). Migration experiments revealed that UVA irradiation significantly impaired cell migration capacity, as evidenced by a reduced wound closure rate. In contrast, GS5 sericin treatment effectively restored cell migration, exhibiting a more pronounced protective effect than WT sericin or Seq10 alone (Figs. 5F**-**G). UVA irradiation also caused a significant increase in ROS levels, which were notably reduced in the GS5 sericin-treated group (Figs. 5H**-**I). Furthermore, UVA-induced DNA damage, as indicated by elevated γ-H2Ax levels, was significantly reduced in the GS5 sericin-treated group (Figs. 5J**-**K). Concurrently, both protein and mRNA levels of inflammatory factors markedly increased following UVA irradiation, with the ameliorative effect of GS5 sericin being the most pronounced (Figs. 5L**-**M). These findings indicate that UVA irradiation enhances epidermal proliferation, senescence, migration, ROS generation, DNA damage, and inflammatory responses. While WT, Seq10, and GS5 all alleviated these effects, GS5 exhibited the strongest protective efficacy from UVA-induced photoaging.

### 3.5 GS5 sericin protects fibroblasts from UVA-induced cellular senescence

**Figure 6:**
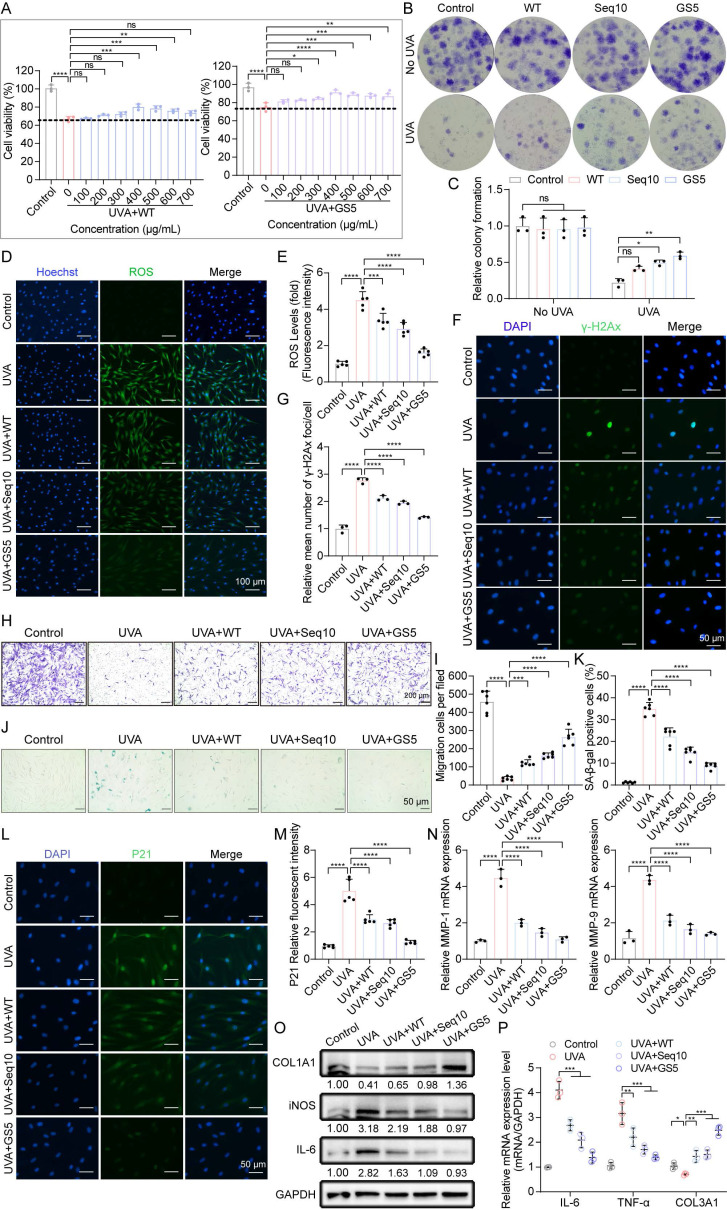
GS5 sericin protects dermal fibroblasts from UVA-induced cellular senescence *in vitro*. (A) The effect of WT or GS5 sericin treatment on the viability of HDFs after UVA irradiation. (B) Representative images of HDFs colonies generated in the control group and radiation groups. (C) Quantification of relative colony formation. (D) Representative immunofluorescence staining images of ROS (green) and Hoechst. Scale bar, 100 μm. (E) Fluorescent intensity of ROS levels. (F) Representative immunofluorescence staining images of γ-H2Ax (green) and DAPI positive cells. Scale bar, 50 μm. (G) Quantification of the average number of γ-H2Ax foci per cell. (H) Representative image of Transwell assay for HDFs. Scale bar, 200 μm. (I) Quantification of the Transwell assay for HDFs. (J) Representative image of SA-β-gal staining in HDFs. Scale bar, 50 μm. (K) Quantification of SA-β-gal positive cells in HDFs. (L) Representative immunofluorescence staining images of P21 (green) and DAPI positive cells. Scale bar, 50 μm. (M) Quantification of relative fluorescence of P21. (N) Quantification of MMP-1 and MMP-9 released by HDFs through qRT-PCR. (O) Western blot analysis showing the change of COL1A1, iNOS and IL-6 in HDFs. (P) Quantification of IL-6, TNF-α, and COL3A1 released by HDFs through qRT-PCR. All data are presented as means ± standard deviation (SD) (n = 3). Statistical significance is denoted by * P < 0.05, ** P < 0.01, *** P < 0.001, and ****P < 0.0001 vs. UVA-irradiated group.

UVA directly penetrates the dermis, where human dermal fibroblasts (HDFs) regulate MMPs/collagen production critical for ECM homeostasis 49. Under physiological conditions, dermal cell proliferation and migration are essential for maintaining skin homeostasis. The CCK8 assay demonstrated that both WT and GS5 exhibited cytotoxicity-free profiles for HDFs (Fig. S9C**-**D). Similar to HaCaTs, HDFs began to take up FITC-labeled GS5 within 2 h of treatment, and the internalized GS5 remained detectable within the cells for up to 12 h (Fig. S11). UVA irradiation significantly inhibited cell proliferation, which was ameliorated by GS5 treatment. Compared to WT, GS5 demonstrated superior efficacy, achieving maximum recovery of proliferation at 400 µg/mL (Fig. 6A). This concentration was selected for subsequent experimental. Colony formation assays further confirmed that GS5 sericin exhibited stronger proliferative effects (Figs. 6B**-**C). Similar to HaCaTs, UVA irradiation increased ROS levels (Figs. 6D**-**E) and induced DNA damage (Figs. 6F**-**G) in HDFs, which were all alleviated by GS5 treatment. Transwell assays revealed that GS5 treatment significantly enhanced cell migration (Figs. 6H**-**I). UVA irradiation also increased the accumulation of SA-β-gal-positive cells and the aging marker P21, both of which were significantly reduced by GS5 treatment (Figs. 6J**-**M). MMPs play a crucial role in maintaining ECM homeostasis. We found that UVA irradiation stimulated the secretion and expression of MMPs, particularly MMP-1 and MMP-9, while simultaneously reducing the levels of COL1A1 and COL3A1, indicating a disruption of collagen homeostasis and degradation of collagen fibers. In contrast, GS5 treatment restored the balance of MMP-1, MMP-9, COL1A1, and COL3A1 levels (Figs. 6N-P). Additionally, inflammatory factors significantly increased after UVA irradiation, and GS5 treatment was also most effective in alleviating the inflammatory response (Figs. 6O-P). These results indicate that UVA irradiation induces ROS, DNA damage, senescence, inflammation, and impaired collagen synthesis in HDFs, the main features of photoaging in the dermis. Remarkably, treatment with GS5 demonstrates a capacity to mitigate these effects. Collectively, our *in vitro* study demonstrates that GS5 is more effective than WT and Seq10 in protecting skin cells from UVA-induced photoaging by regulating various cellular processes.

### 3.6 Nrf2 is essential for GS5 sericin to exert its anti-photoaging effect

**Figure 7:**
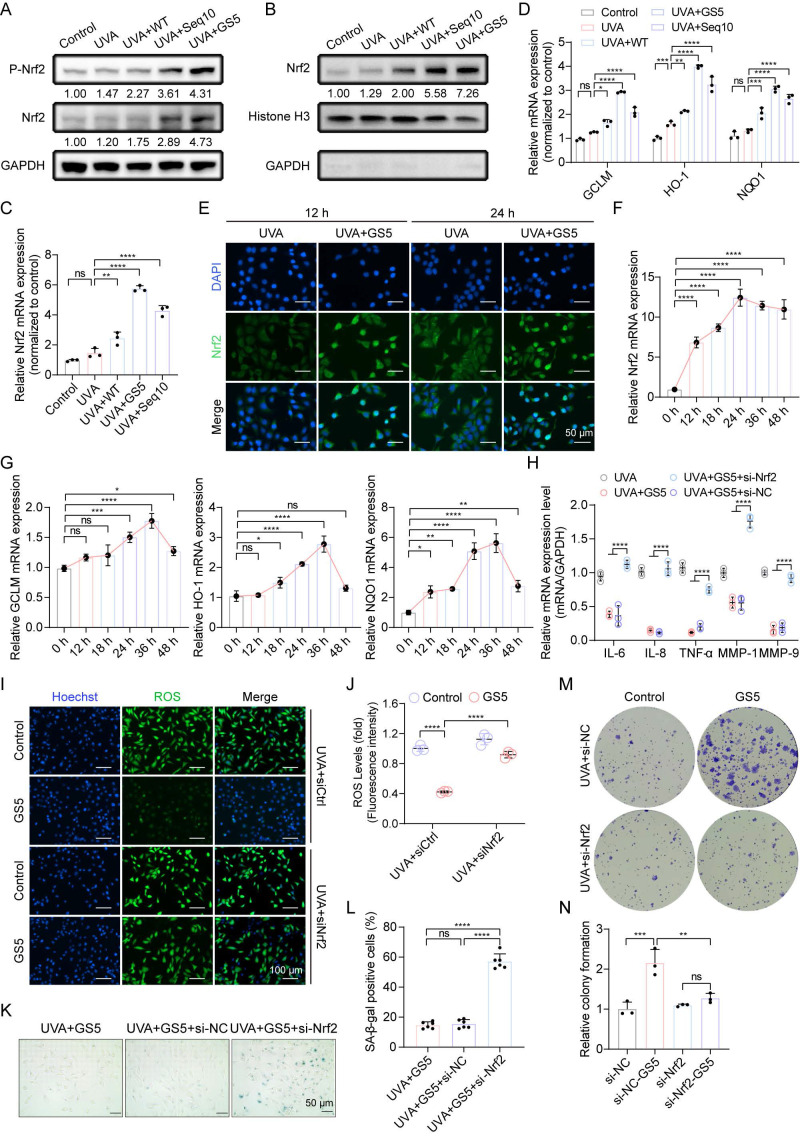
Nrf2 is essential for the anti-photoaging effect and SASP of GS5. (A) Western blot analysis for Nrf2 and P-Nrf2 expression levels in whole cell lysates. (B) Western blot analysis for Nrf2 expression levels in the nucleus. (C**-**D) Relative mRNA expression of Nrf2, GCLM, HO-1, and NQO1 in the control group and UVA radiation group. (E) Immunofluorescence images of Nrf2 (green) at 12 h and 24 h in each group. Scale bar, 50 μm. (F**-**G) Changes in relative mRNA levels of Nrf2, GCLM, HO-1, and NQO1 in irradiated HaCaTs treated with GS5 over time. (H) Quantification of mRNA secretion of IL-6, IL-1β, TNF-α, MMP-1, and MMP-9 in HaCaTs after Nrf2 knockdown. (I) Analysis of ROS (green) levels in GS5-treated cells after Nrf2 knockdown. Scale bar, 100 µm. (J) Fluorescence intensity of ROS levels. (K) Representative images of SA-β-gal staining in HaCaTs after Nrf2 knockdown. Scale bar, 50 μm. (L) Quantification of SA-β-gal positive cells. (M) Representative images of cell colonies formed in the survival assay after Nrf2 knockdown. (N) Quantification of cell colony formation. All data are presented as means ± standard deviation (SD) (n = 3). Statistical significance is denoted by * P < 0.05, ** P < 0.01, *** P < 0.001, and ****P < 0.0001 vs. UVA-irradiated group.

Aforementioned studies have shown that Seq10 activates Nrf2 to mitigate photoaging, which motivated us to investigate whether GS5 sericin regulates Nrf2 activation in HaCaTs. We found that GS5 sericin treatment maximally increased the expression of Nrf2 in HaCaTs, and its phosphorylation level also significantly rose, indicating the activation of the Nrf2 pathway (Fig. 7A). The activation of Nrf2 was further confirmed by its translocation to the nucleus. Western blot analysis of nuclear extracts revealed that GS5 sericin treatment induced a substantial accumulation of Nrf2 in the nuclear fraction (Fig. 7B). Immunofluorescence staining confirmed that in GS5 sericin-treated cells, the nuclear localization of Nrf2 increased, peaking at 24 h (Fig. 7E). We subsequently measured the relative expression of Nrf2 and downstream target genes GCLM, HO-1, and NQO1 in the cells using qRT-PCR. Compared to other treatment groups, GS5 sericin treatment resulted in the greatest upregulation of these genes (Figs. 7C-D). Additionally, after GS5 sericin treatment, Nrf2 reached its highest level at 24 h, while its downstream antioxidant target genes peaked at 36 h (Figs. F-G). These findings suggest that GS5 sericin activates Nrf2 to protect HaCaTs from UVA-induced oxidative stress. To confirm the role of the Nrf2 pathway in the protective effects of GS5 sericin, HaCaTs were transfected with si-Nrf2 or si-NC. The results showed that Nrf2 knockdown abolished the ability of GS5 sericin to reduce the expression of SASP-related genes (IL-6, IL-8, TNF-α, MMP-1, and MMP-9) and ROS production induced by UVA (Fig. 7H**-**J). Additionally, the effect of GS5 sericin in reducing the proportion of SA-β-gal positive cells was largely lost (Figs. 7K-L), and the proliferative ability originally possessed by GS5 sericin was significantly weakened (Fig. 7M-N). These findings conclusively demonstrate that Nrf2 is a key molecular target for the action of GS5 sericin and is crucial for its photoprotective effect.

### 3.7 The therapeutic effect of GS5 sericin in a mouse model of photoaging

**Figure 8:**
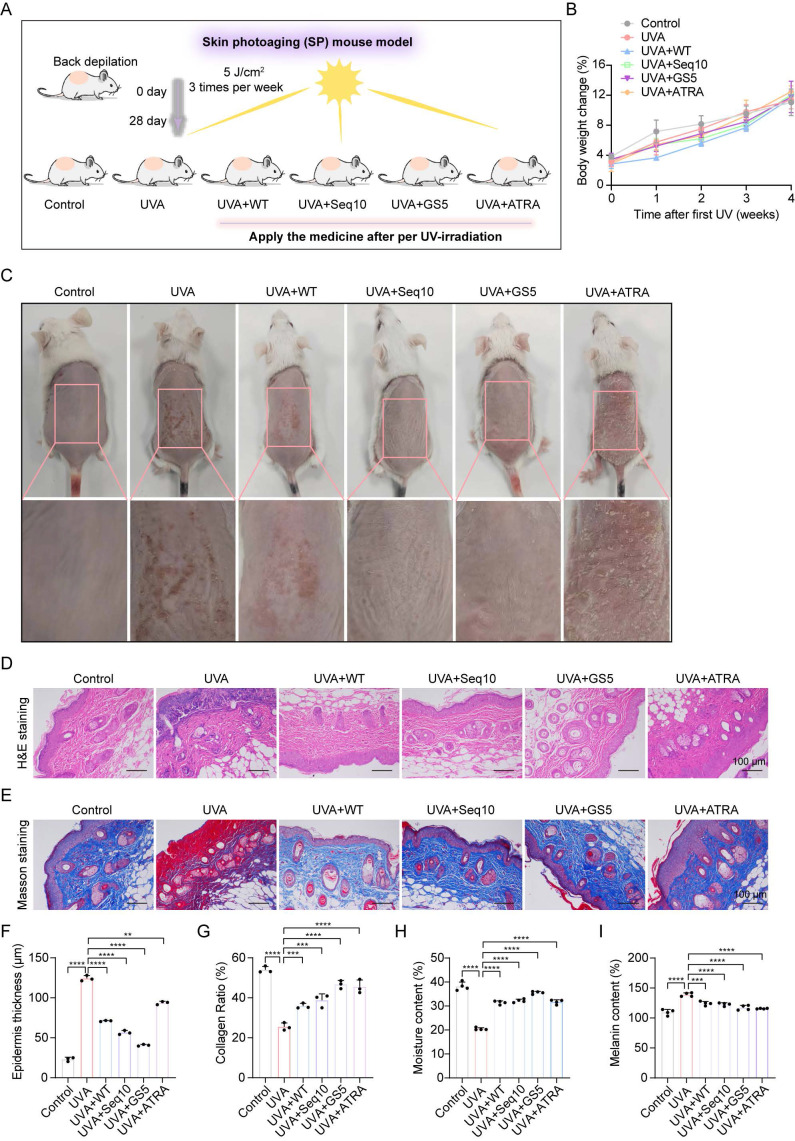
Histological analysis of dorsal skin in mice after UVA irradiation and GS5 sericin treatment. (A) Schematic representation of the establishment and treatment of the mouse photoaging model. (B) Changes in body weight of mice during UVA radiation. (C) Representative images of mouse dorsal skin. From left to right: control group (PBS treatment), UVA radiation groups treated with PBS, WT sericin, Seq10, GS5 sericin, and positive control ATRA. (D) Representative images of H&E staining. Scale bar, 100 μm. (E) Representative images of Masson staining. Scale bar, 100 μm. (F) Analysis of epidermal thickness. (G) Quantification of collagen area from Masson staining statistics. (H) Amount of transepidermal water loss. (I) Melanin content in skin. All data are presented as means ± standard deviation (SD) (n = 3). Statistical significance is denoted by * P < 0.05, ** P < 0.01, *** P < 0.001, and ****P < 0.0001 vs. UVA-irradiated group.

**Figure 9:**
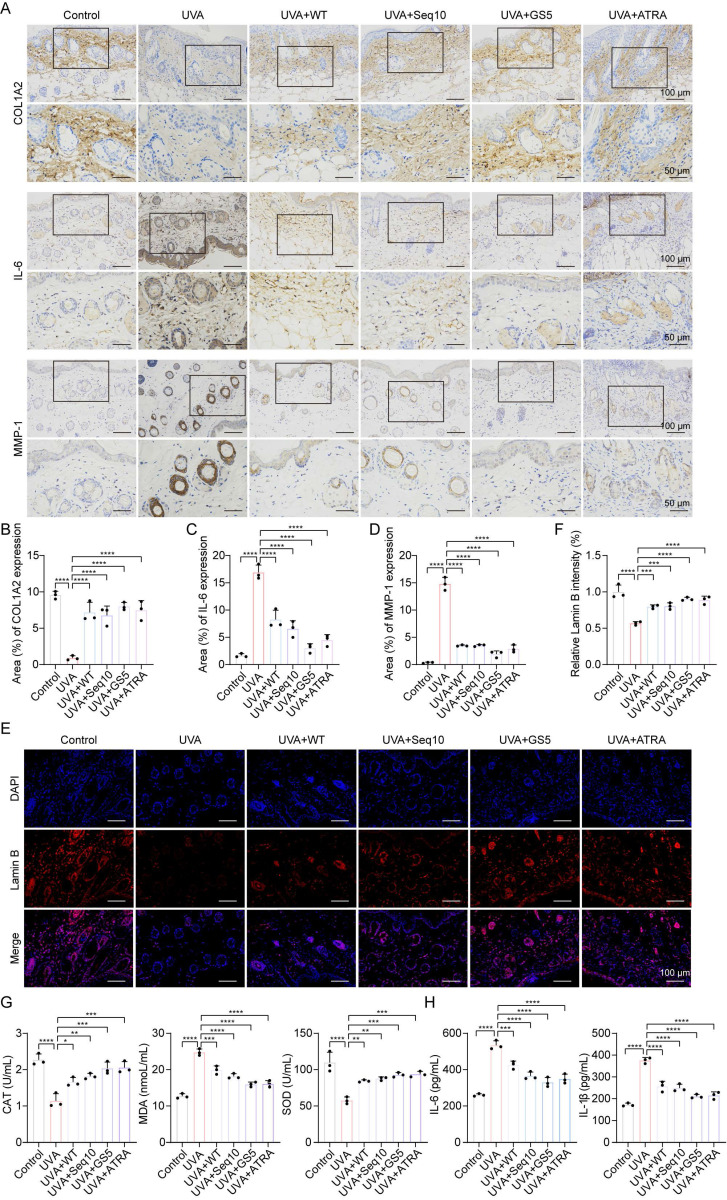
GS5 sericin alleviates UVA-induced skin photodamage in mice. (A) Immunohistochemical staining of COL1A2, IL-6, and MMP-1 in the dorsal skin of mice. Scale bar, 50 μm. (B) Quantification of COL1A2 by qRT-PCR. (C) Quantification of IL-6 by qRT-PCR. (D) Quantification of MMP-1. (E) Immunofluorescence staining of Lamin B (red) in the dorsal skin of mice. Scale bar, 100 μm. (F) Quantification of Lamin B positive cells. (G) Quantification of CAT, MDA, and SOD in the skin. (H) Quantification of IL-6 and IL-1β released from the skin measured by ELISA. All data are presented as means ± standard deviation (SD) (n = 3). Statistical significance is denoted by * P < 0.05, ** P < 0.01, *** P < 0.001, and ****P < 0.0001 vs. UVA-irradiated group.

The promising *in vitro* results of GS5 sericin prompted us to investigate its effects on skin photoaging *in vivo*. A photoaging model was established to evaluate the therapeutic effects of GS5 sericin (Fig. 8A). Body weight measurements showed no significant changes across treatment groups, confirming the safety of the interventions (Fig. 8B). At week 4, dorsal skin images revealed that the UVA-treated group exhibited deeper and wider wrinkles, along with severe erythema and desquamation, successfully establishing the photoaging model (Fig. 8C). In contrast, the GS5 sericin-treated group demonstrated significant reductions in wrinkles, erythema, and desquamation compared to other treatment groups. Histological analysis revealed that UVA irradiation increased epidermal thickness and decreased dermal collagen compared to the control group (Figs. 8D**-**G). However, GS5 sericin treatment effectively restored epidermal thickness and increased dermal collagen levels, surpassing the therapeutic effects of the positive control ATRA (Figs. 8D**-**G). Additionally, GS5 sericin-treated skin exhibited the highest moisture content and the greatest reduction in UVA-induced melanin accumulation compared to other treatment groups (Figs. 8H**-**I). Immunohistochemistry demonstrated that UVA radiation increased IL-6 and MMP-1 expression while decreasing COL1A2 expression. GS5 sericin treatment effectively counteracted these changes, showing superior therapeutic effects compared to other treatment groups (Figs. 9A**-**D). Immunofluorescence staining further confirmed that GS5 sericin maximally restored the aging marker Lamin B (Figs. 9E**-**F). Biochemical assays revealed that GS5 sericin and ATRA maximally reversed UVA-induced reductions in CAT and SOD levels and increases in MDA levels (Fig. 9G). ELISA results showed that GS5 sericin most effectively restored UVA-induced increases in inflammatory factors IL-6 and IL-1β (Fig. 9H). Overall, these findings demonstrate that GS5 sericin reverses UVA-induced skin changes, including oxidative damage, inflammatory responses, and histological alterations, with superior therapeutic effects compared to other treatments.

### 3.8 Transcriptomics-based identification of the potential molecular mechanism of GS5 sericin in improving cellular photoaging

**Figure 10:**
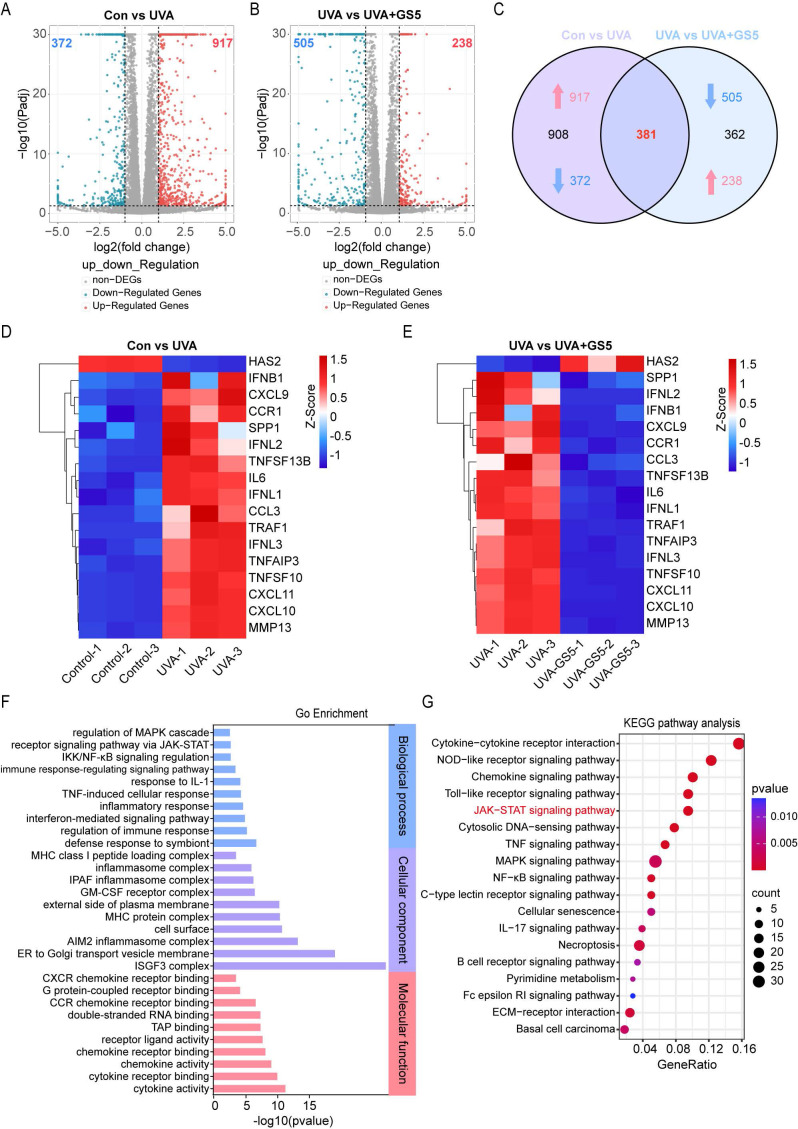
Identification of potential molecular mechanisms by which GS5 improves cellular photoaging based on transcriptomics. (A**-**B) Volcano plots showing the differentially expressed genes between the Con vs UVA and UVA vs UVA+GS5 groups. (C) Venn diagram illustrating the overlap between upregulated and downregulated genes. (D**-**E) Heatmap analysis of differentially expressed SASP genes in the Con vs UVA and UVA vs UVA+GS5 groups. (F) GO enrichment analysis of overlapping differentially expressed genes, including biological processes (blue), cellular components (purple), and molecular functions (red). (G) Bubble plot of KEGG pathway analysis. All data are presented as means ± standard deviation (SD) (n = 3). Statistical significance is denoted by * P < 0.05, ** P < 0.01, *** P < 0.001, and ****P < 0.0001 vs. UVA-irradiated group.

To investigate the mechanisms underlying the anti-photoaging effects of GS5 sericin, we pretreated HaCaTs with GS5 sericin for 24 h followed by continuous UVA irradiation for 2 days. RNA-seq analysis revealed differential gene expression between treatment groups. Compared to the control group, the UVA group exhibited 372 downregulated and 917 upregulated genes (|log2(FC)| > 1, P value < 0.05) (Fig. 10A). Between the UVA and UVA+GS5 groups, 505 genes were downregulated and 238 genes were upregulated (|log2(FC)| > 1, P value < 0.05) (Fig. 10B). Of these, 381 genes were either upregulated by UVA and inhibited by GS5 sericin or downregulated by UVA and restored by GS5 sericin (Fig. 10C). Notably, GS5 sericin modulated UVA-induced changes in SASP-related genes, downregulating those that were upregulated by UVA and upregulating those that were downregulated (Figs. 10D**-**E). GO analysis revealed that these differentially expressed genes are associated with immune regulation and inflammatory responses (Fig. 10F). KEGG pathway analysis further highlighted enrichment in the JAK**-**STAT and inflammatory signaling pathways (Fig. 10G).

**Figure 11:**
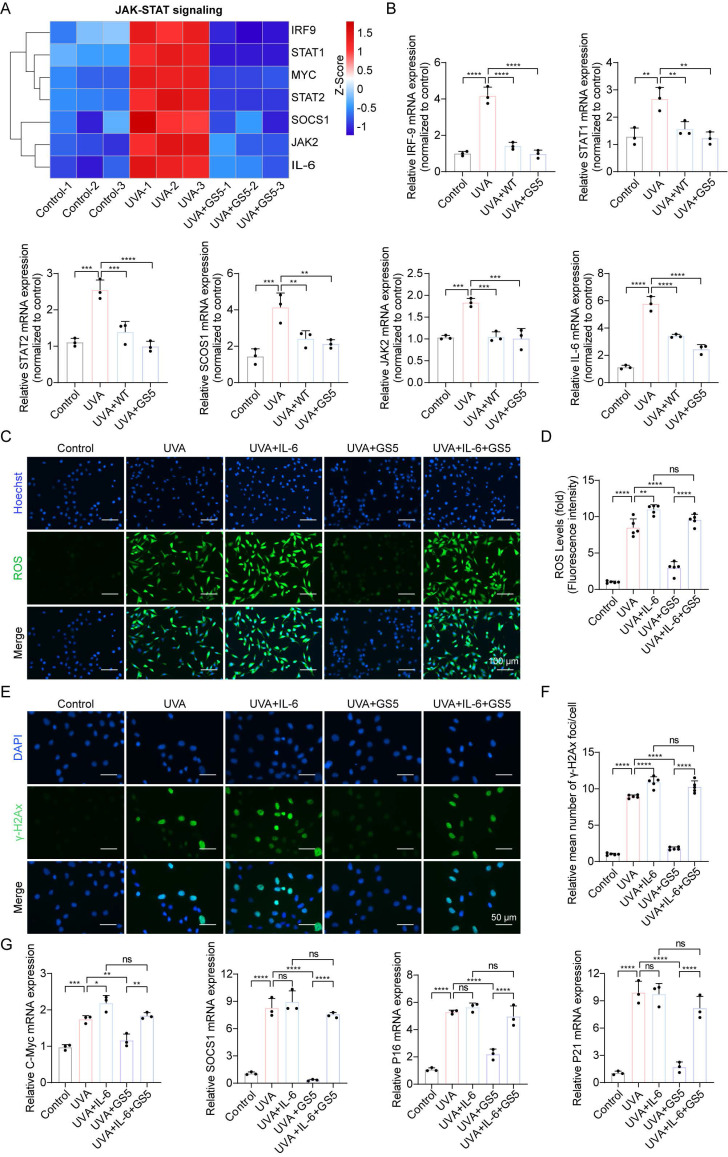
GS5 also rescues UVA-induced cellular photoaging by downregulating JAK-STAT signaling. (A) A heatmap showing the gene expression profile in response to UVA and GS5 sericin treatment in JAK-STAT signaling. (B) Quantification of different mRNA levels of IRF-9, STAT1, STAT2, SCOS1, JAK2, and IL-6. (C) Representative immunofluorescence staining images of ROS after IL-6 and GS5 treatment. Scale bar, 100 µm. (D) Fluorescent quantification of ROS levels. (E) Representative immunofluorescence staining images of γ-H2Ax after IL-6 and GS5 treatment. Scale bar, 50 µm. (F) Quantification of the average number of γ-H2Ax foci per cell. (G) Quantification of different mRNA levels of C-Myc, SOCS1, P16, and P21 after IL-6 and GS5 treatment. All data are presented as means ± standard deviation (SD) (n = 3). Statistical significance is denoted by * P < 0.05, ** P < 0.01, *** P < 0.001, and ****P < 0.0001 vs. UVA-irradiated group.

Previous studies have shown that JAK**-**STAT pathway activation accelerates UVB-induced photodamage 50. In this study, GS5 sericin downregulated UVA-induced genes in the JAK**-**STAT signaling pathway (Fig. 11A). qPCR confirmed the changes in key JAK**-**STAT pathway molecules, including IRF-9, STAT1, STAT2, SCOS1, JAK2, and IL-6 (Fig. 11B). The JAK**-**STAT pathway regulates cell growth, differentiation, apoptosis, and immune responses, particularly through IL-6 signaling 51, 52. To determine whether GS5 sericin regulates downstream pathways by inhibiting JAK**-**STAT signaling, we used IL-6, a JAK**-**STAT pathway activator. Addition of IL-6 to HaCaTs reduced the efficacy of GS5 sericin in clearing ROS and repairing DNA damage (Figs. 11C**-**F). c-Myc and SOCS1 are key downstream molecules of the JAK**-**STAT pathway. Since p16 and p21 are established UVA-induced senescence markers, and JAK**-**STAT activation promotes ROS/inflammatory microenvironments that upregulate these markers 53. qPCR revealed GS5 reduced efficacy in suppressing c-Myc, SOCS1, p16, and p21 expression under IL-6 stimulation (Fig. 11G). In summary, these findings suggest that may exert its photoprotective effects by inhibiting the IL-6-mediated JAK**-**STAT signaling.

## 4. Discussion and Conclusion

UVA radiation and HEV light are primary exogenous drivers of skin photoaging. Both penetrate the skin to induce oxidative stress, inflammation, immune suppression, and extracellular matrix degradation through overlapping pathways 54. Therefore, strategies targeting shared molecular mechanisms, such as the activation of antioxidant defenses, are crucial for comprehensive photoprotection. Current therapeutic strategies, including retinoids, sunscreens, and antioxidants, are limited by adverse effects, biosafety concerns, environmental impact, and poor photostability. Additionally, most research focuses on single pathways, neglecting the complex signaling networks that drive photoaging, which undermines therapeutic efficacy. These limitations highlight the urgent need for safer and more effective treatments. Nrf2, a master regulator of antioxidant defense, mitigates oxidative stress by activating downstream protective genes, representing a promising nodal target against insults from both UVA and HEV light 55. However, conventional small-molecule Nrf2 activators are plagued by off-target effects and dose-limiting toxicity 56.

This study targets the Keap1**-**Nrf2 PPI and identifies Seq10, an effective PPI inhibitor, through molecular docking and molecular dynamics simulations. Seq10 forms a stable complex with Keap1 via van der Waals forces, hydrogen bonds, and other interactions, as evidenced by its low fluctuation and stable binding conformations during dynamic simulations. *In vitro* experiments demonstrate that Seq10 binds to Keap1, activates Nrf2 transcriptional activity, and significantly alleviates UVA-induced cellular photoaging. Importantly, Nrf2 gene knockdown experiments confirm that the anti-photoaging effect of Seq10 is Nrf2-dependent, underscoring the critical role of Nrf2 in its protective effects. Since Nrf2 activation is a validated defense mechanism against HEV light-induced oxidative stress 57, the efficacy of Seq10 suggests its potential utility beyond UVA protection.

However, peptide molecules pose challenges such as low dermal penetration efficiency and short *in vivo* half-life, which limit their translational application in anti-photoaging research. To address these limitations, we employed sericin, a natural glycoprotein abundant in hydrophilic groups derived from silkworm silk, as a drug carrier. Sericin exhibits excellent biocompatibility, water solubility, moisturizing properties, and intrinsic antioxidant activity, making it an ideal candidate for anti-photoaging studies 58. This study ingeniously integrated Seq10 with sericin to develop a novel GS5 sericin, which demonstrated superior anti-photoaging functionality. To achieve robust expression of GS5, we optimized the gene sequence and successfully expressed the recombinant GS5 protein in transgenic silkworms. GS5 sericin with high antioxidant activity was obtained via enzymatic hydrolysis and ultrafiltration. *In vitro* cell experiments revealed that GS5 sericin exhibited exceptional anti-photoaging effects. For HaCaTs, it significantly ameliorated UVA-induced decreases in cell viability, migration inhibition, elevated ROS levels, DNA damage, and inflammatory responses. In HDFs, GS5 sericin effectively promoted cell proliferation, inhibited aging, reduced ROS production and DNA damage, regulated MMPs secretion and inflammatory responses, and restored collagen homeostasis. These results demonstrate that GS5 sericin provides robust protection for both epidermal and dermal cells, comprehensively mitigating UVA-induced cellular photoaging, with superior efficacy compared to WT sericin and Seq10 alone.

Further investigations demonstrate that Nrf2 is a critical mediator of GS5 sericin's anti-photoaging effects. Our data demonstrated that Seq10 and its sericin-fusion form GS5 significantly enhanced Nrf2 phosphorylation through binding with Keap1, thereby promoting its release from the cytoplasmic complex, nuclear translocation, and subsequent transcriptional activation of antioxidant genes. Validation via Nrf2 knockdown experiments showed that the anti-photoaging effects of GS5 sericin were markedly diminished, failing to reduce UVA-induced ROS production and SASP expression, or restore cell clonogenicity and SA-β-gal positive cell reversal. These findings confirm that GS5 sericin primarily exerts its anti-photoaging effects through Nrf2 pathway activation, regulating antioxidant and anti-inflammatory responses. This mechanism is highly relevant for HEV light protection, as Nrf2 activation has been shown to ameliorate blue light-induced skin damage 59.

*In vivo* experiments using a mouse photoaging model demonstrated that GS5 sericin exhibited potent therapeutic effects. It significantly reduced wrinkle formation, erythema, and desquamation in mouse skin, decreased epidermal thickness, and increased dermal collagen content, thereby improving skin appearance and tissue structure. Additionally, GS5 sericin enhanced skin moisture, reduced melanin levels, regulated protein expression, and reversed UVA-induced skin damage. Its therapeutic effects surpassed those of other treatment groups and even outperformed the positive control, ATRA, further confirming the efficacy and superiority of GS5 sericin in anti-photoaging *in vivo*.

Furthermore, RNA-seq analysis revealed the molecular mechanism underlying GS5 sericin's ability to ameliorate cellular photoaging. The study demonstrated that GS5 sericin regulates the expression of multiple genes involved in immune and inflammatory responses, significantly enriching the JAK-STAT and related inflammatory signaling pathways. Specifically, GS5 sericin was found to downregulate UVA-induced gene expression within the JAK-STAT pathway. Crucially, when the pathway was activated by IL-6, the efficacy of GS5 sericin in clearing ROS, repairing DNA damage, and reducing aging markers was diminished. Collectively, these findings suggest that GS5 sericin mitigates photoaging primarily by inhibiting the IL-6-mediated JAK-STAT signaling pathway, which in turn regulates cellular processes such as growth, differentiation, apoptosis, and immune homeostasis. Notably, the JAK-STAT pathway is also involved in oxidative stress responses, a key component of HEV light damage 60. Thus, this study provides a theoretical foundation for applying GS5 sericin against photoaging induced by multiple wavelengths of solar radiation.

Compared with conventional photoprotective therapies, the strategy integrating peptide inhibitors targeting PPI with natural sericin offers significant advantages, including enhanced drug targeting, biocompatibility, and safety. The production of these agents using silkworm gland bioreactors is environmentally sustainable and cost-effective. Furthermore, this study provides comprehensive evidence, validated at the molecular, cellular, and animal levels, for the anti-photoaging efficacy of GS5 sericin.

This research not only proposes novel concepts and strategies for treating skin photoaging but also expands the potential applications of natural proteins in biomedicine. By demonstrating the efficacy of GS5 against UVA through the Nrf2 and JAK-STAT pathways, our work provides a strong rationale for its potential in addressing the challenge of HEV light protection. However, certain limitations remain. For instance, while the well-characterized HaCaT cell line was used here as a standard model, the findings warrant further validation in primary human keratinocytes and a more comprehensive assessment of senescence, such as by detecting lipofuscin accumulation in long-term *in vivo* models, to definitively confirm the anti-senescent efficacy. Additionally, whether GS5 sericin exerts synergistic effects via other signaling pathways or molecular targets requires validation through additional experiments. Future studies could also focus on optimizing the preparation process, enhancing production efficiency, and conducting comprehensive safety and efficacy assessments to advance clinical translation and practical applications in cosmetics and skincare.

## Supplementary Material

Supplementary figures and tables.
