# Supplementary material for: Novel GS5 sericin mitigates UVA-induced photoaging by activating Nrf2 and inhibiting the JAK-STAT pathway
Source: Int J Biol Sci. 2026 Jan 14;22(3):1496–519. doi: 10.7150/ijbs.123702 (PMC12839140; doi:10.7150/ijbs.123702)
Supplement: Supplementary file 1 — Supplementary figures and tables. [file ijbsv22p1496s1.pdf]

Novel GS5 sericin mitigate UVA-induced photoaging by activating Nrf2 and inhibiting the JAK-STAT pathway

Supplementary Figures

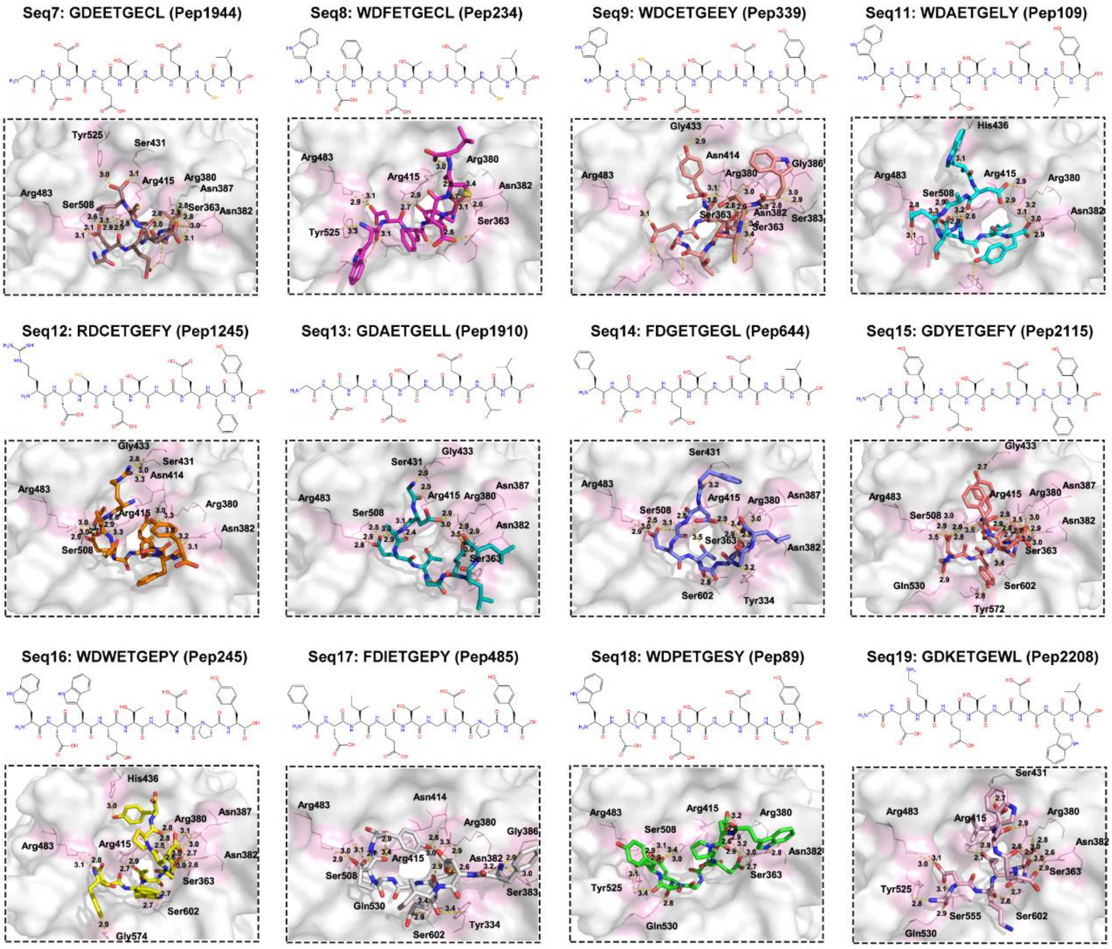

Fig. S1. Visualization of the interactions between the candidate peptide and the Keap1 complex after molecular docking.

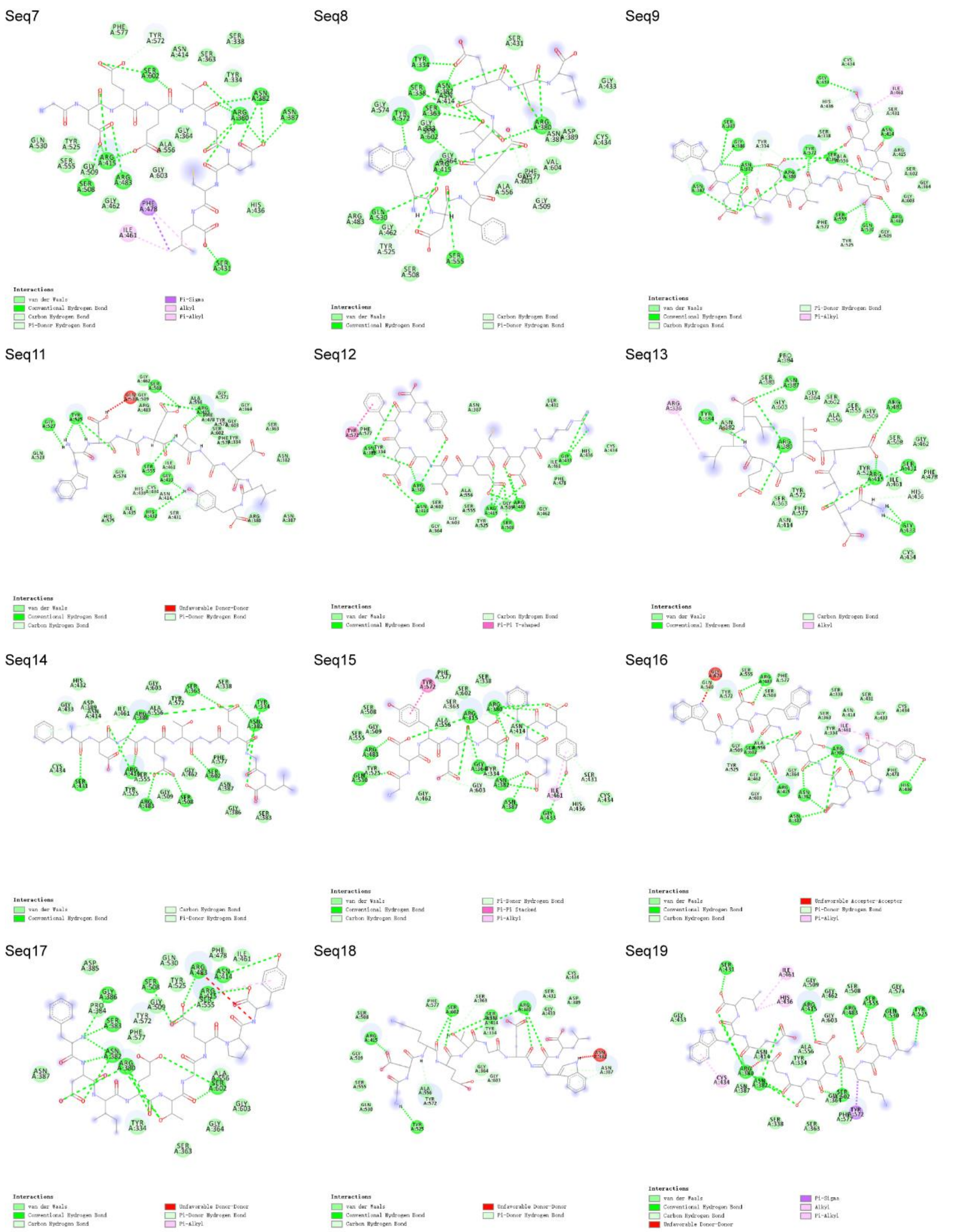

Fig. S2. 2D binding schematic of candidate peptides interacting with Keap1, highlighting the precise non-covalent interactions between them.

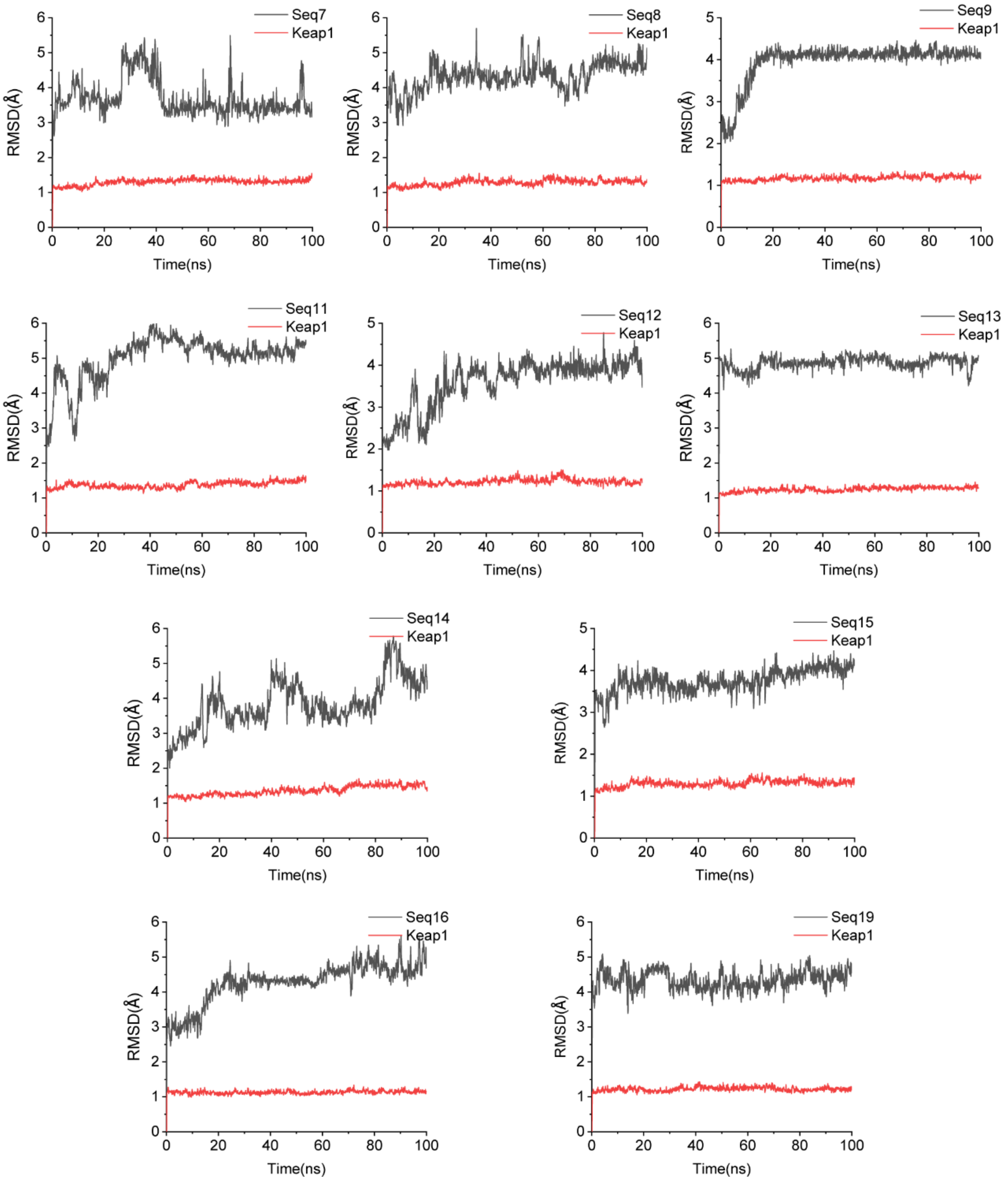

Fig. S3. RMSD curve of the MD simulation of the peptide-Keap1 complex.

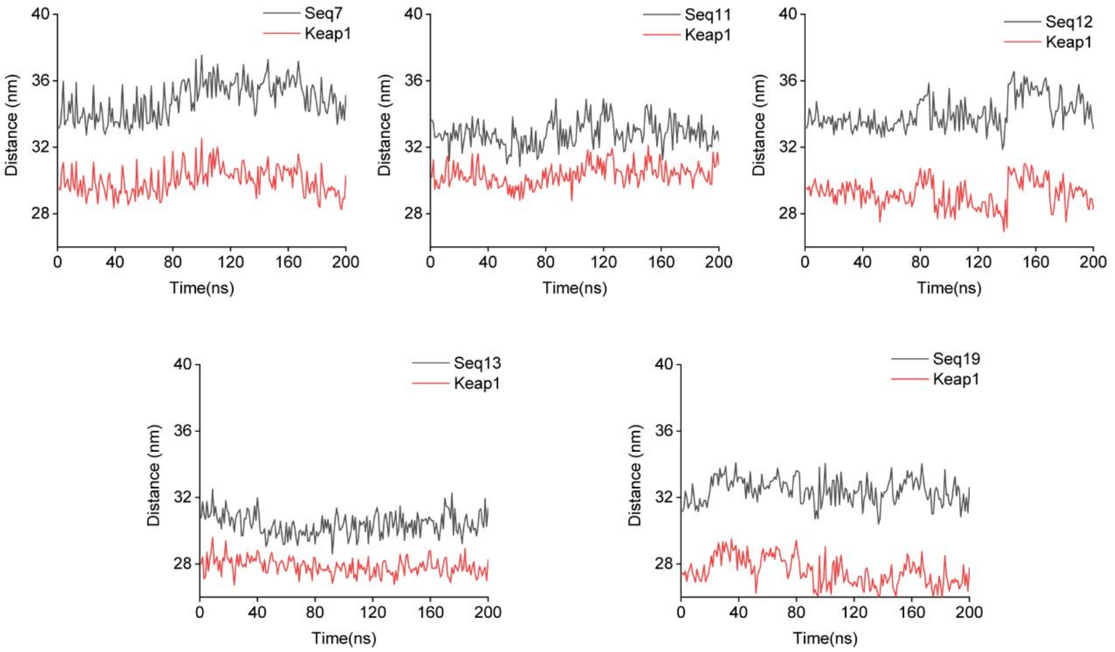

Fig. S4. The variation of distance between the peptide and Keap1 complex over time as observed in MD simulations.

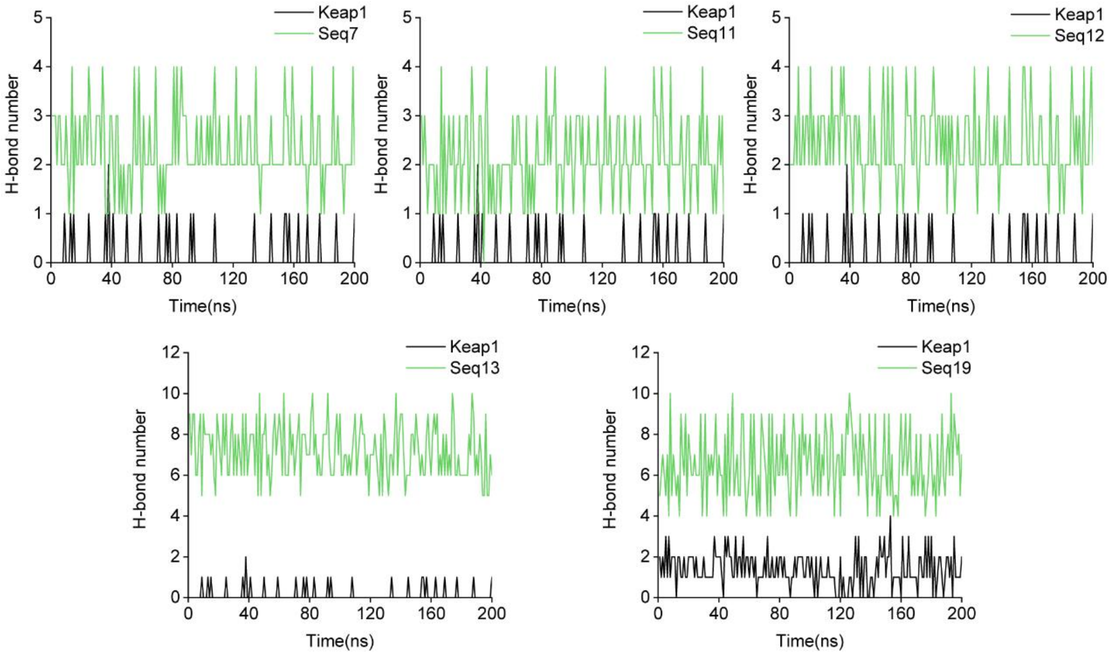

Fig. S5. The variation of hydrogen bonds in the MD simulation of the peptide-Keap1 complex over time.

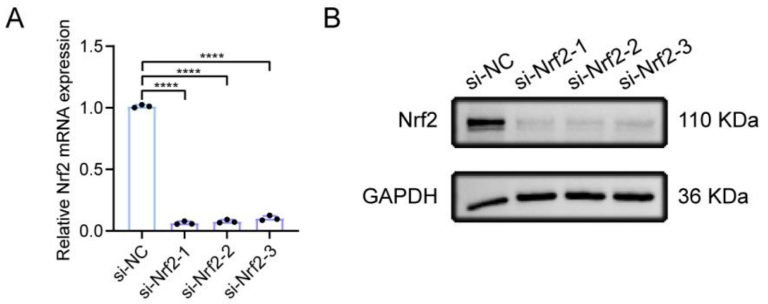

Fig. S6. Verification of Nrf2 Knockdown. (A) Detection at the mRNA level; (B) Detection at the protein level.

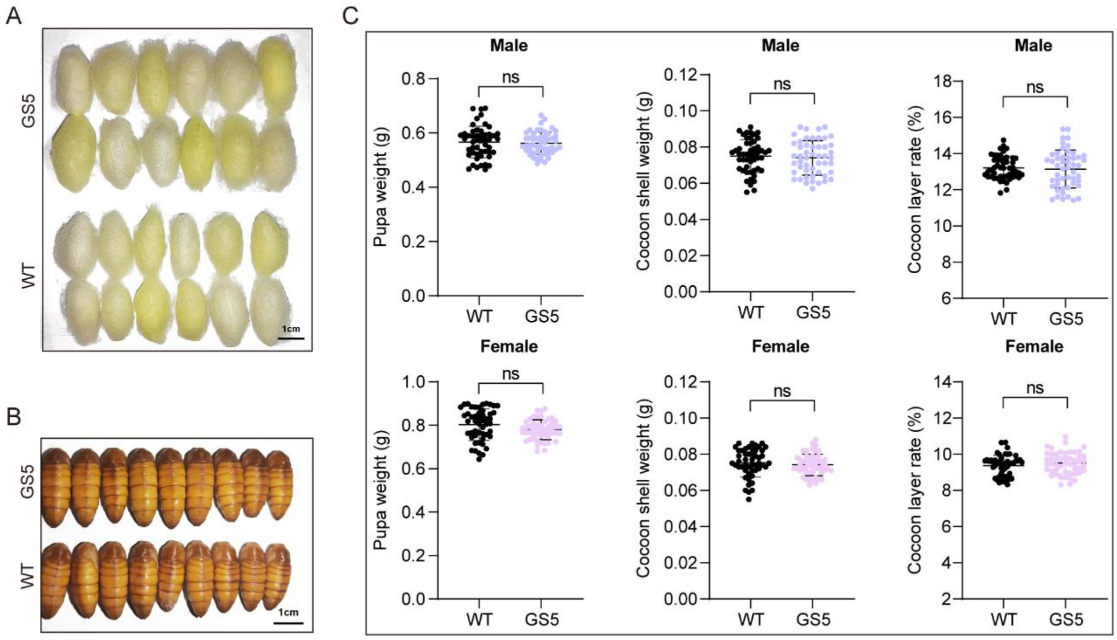

Fig. S7. Economic shape evaluation of transgenic silkworms. (A-B) Representative images of cocoons and pupae from WT silkworms and GS5 transgenic silkworms. (C) Statistical analysis of pupal weight, cocoon layer weight, and cocoon layer rate for male and female GS5 transgenic silkworms.

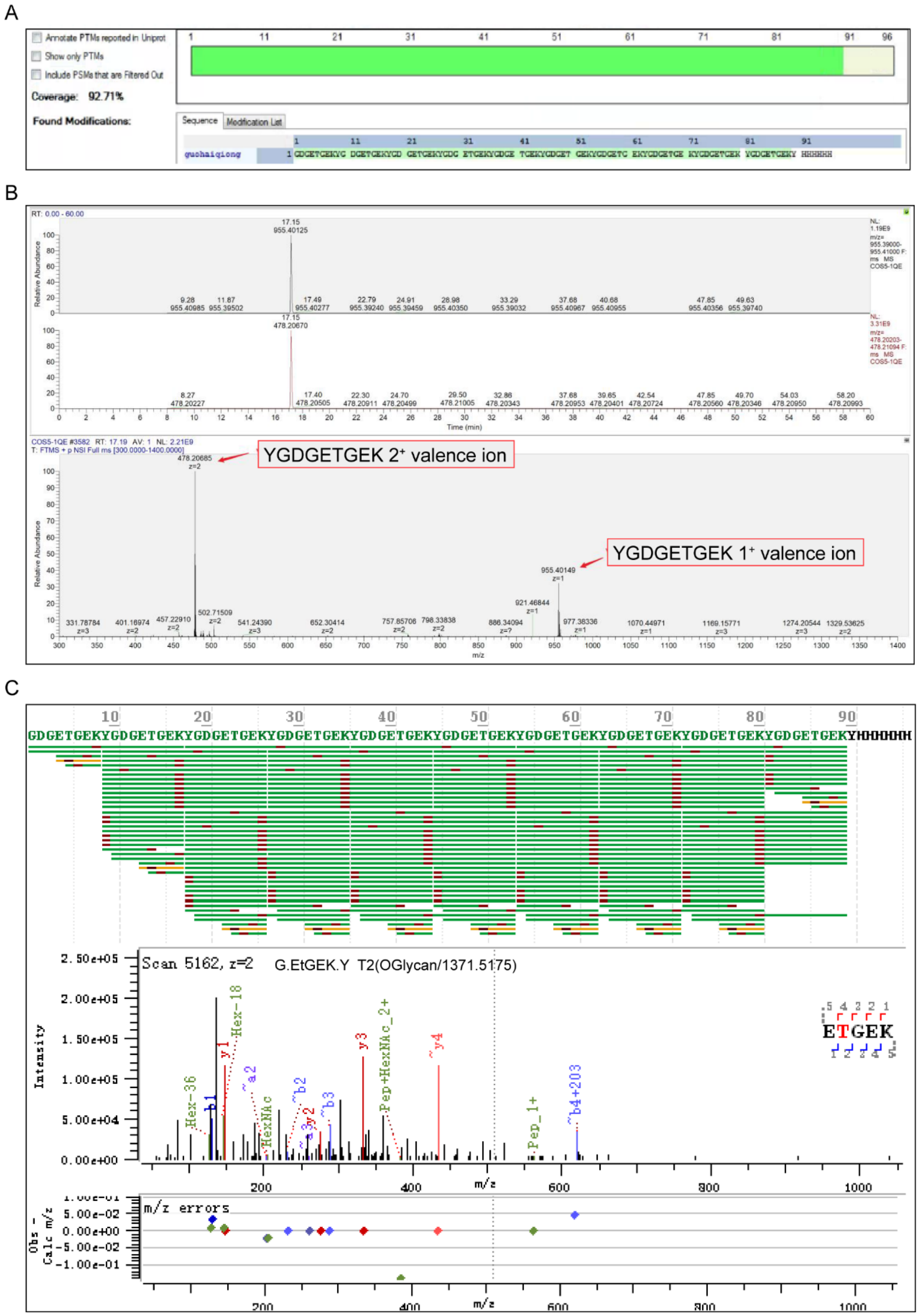

Fig. S8. Mass spectrometric identification of GS5 protein expressed in transgenic silkworms. (A) The amino acid sequence matching degree of the target protein. (B) Mass spectrometry of the characteristic peptide segment of the target protein. (C)

Identification of O-glycosylation modification sites.

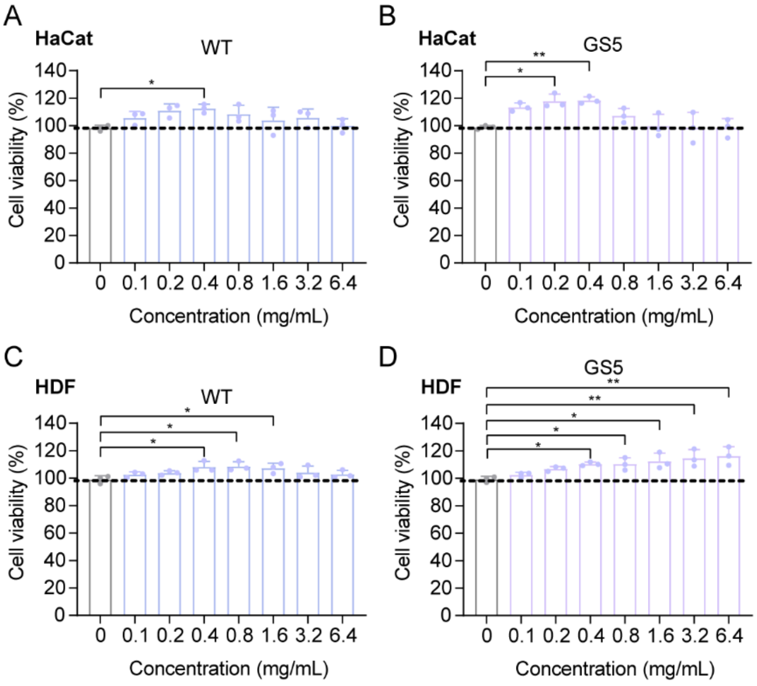

Fig. S9. Effects of WT or GS5 sericin on cell viability. (A-B) Cell viability of HaCaTs treated with specified concentrations of WT or GS5 for 24 h. (C-D) Cell viability of HDFs treated with specified concentrations of WT or GS5 for 24 h.

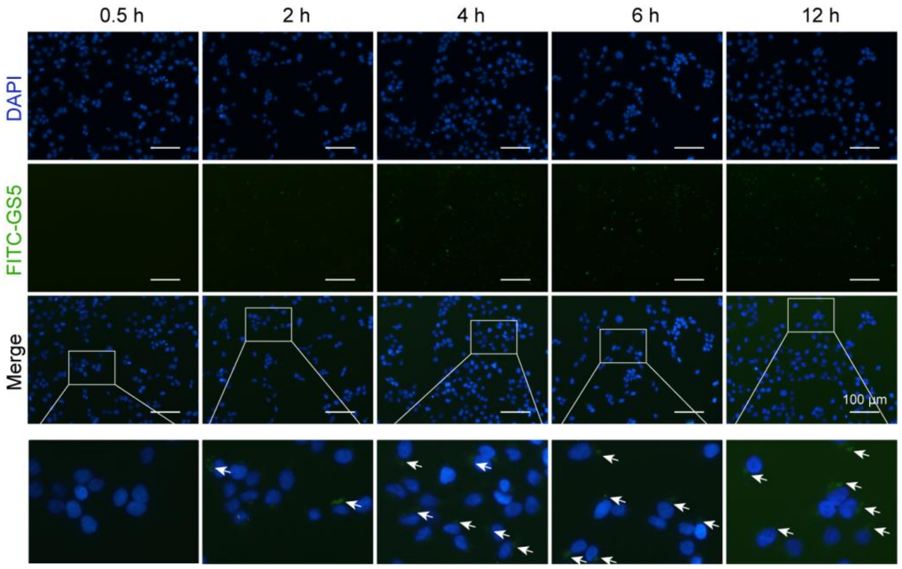

Fig. S10. Representative images of HaCaTs uptake of GS5 at different time points.

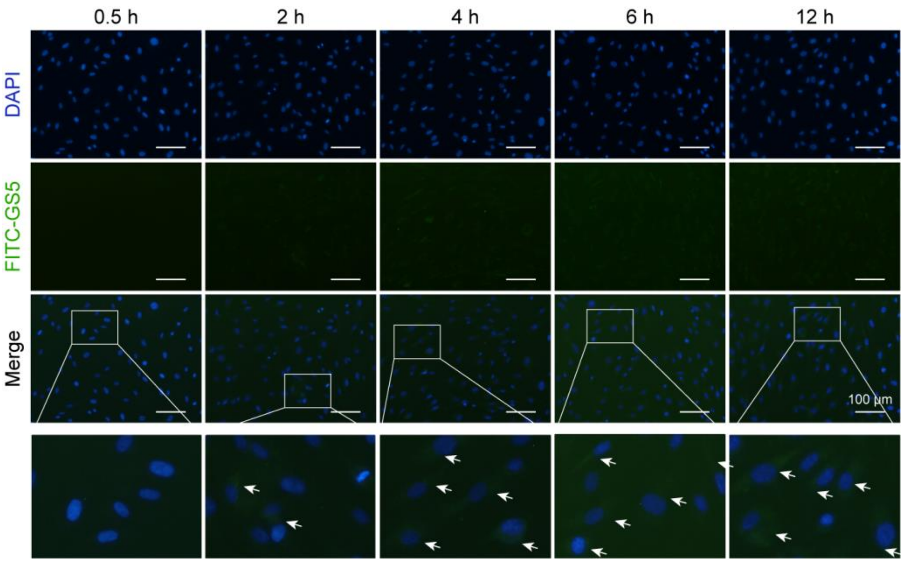

Fig. S11. Representative images of HDFs uptake of GS5 at different time points.

Supplementary Tables

Table S1. Detailed information on High Performance Liquid Chromatography (HPLC) and Mass Spectrometry (MS) of in-house peptides.

Seq. NO.	MW (g/mol)		HPLC purity (%)	Appearance
	Theoretical	Observed		
Seq7	993.9	993.3	98.7	White lyophilized powder
Seq8	1141.2	1140.6	98.0	White lyophilized powder
Seq9	1173.1	1172.8	95.1	White lyophilized powder
Seq10	996.9	996.8	95.9	White lyophilized powder
Seq11	1125.1	1125.0	97.9	White lyophilized powder
Seq12	1161.20	1160.8	97.8	White lyophilized powder
Seq13	945.9	945.6	95.8	White lyophilized powder
Seq14	965.9	965.2	98.3	White lyophilized powder
Seq15	1122.10	1121.6	98.9	White lyophilized powder
Seq16	1224.2	1224.8	98.4	White lyophilized powder
Seq17	1112.1	1111.2	97.5	White lyophilized powder
Seq18	1125.1	1124.4	96.0	White lyophilized powder
Seq19	1076.1	1076.8	96.6	White lyophilized powder
Seq20	1078.1	1077.6	97.6	White lyophilized powder

MW: molecular weight.

Table S2. Sequences of the siRNA for RNA interference

Gene	Sequence
si-Nrf2-1	F: 5′-CAGUCUUCAUUGCUACUAA-3′
	R: 5′-UUAGUAGCAAUGAAGACUG-3'
si-Nrf2-2	F: 5′-CAUUGAUGUUUCUGAUCUA-3′
	R: 5′-UAGAUCAGAAACAUCAAUG-3'
si-Nrf2-3	F: 5′-GACAGAAGUUGACAAUUAU-3′
	R: 5′-AUAUUAGUCAACUUCUGUC-3'

siRNA: Small interfering RNA

Table S3 Details of antibodies.

Antibodies	Source	Product number
MMP-1	Beyotime, Shanghai, China	AF0231
MMP-9	Beyotime, Shanghai, China	AF5234
GAPDH	Beyotime, Shanghai, China	AF1186
Lamin B1	Beyotime, Shanghai, China	AF1408
IL-1β	Cell Signaling Technology, Danvers, MA, USA	Cat No. 31202
IL-6	Beyotime, Shanghai, China	AF0201
COX2	Beyotime, Shanghai, China	AF1924
iNOS	Beyotime, Shanghai, China	AG4771
Phospho-Nrf2	ABclonal, Wuhan, China	AP1498

Nrf2	Proteintech, Wuhan, China	Cat No. 80593-1-RR
Keap1	Proteintech, Wuhan, China	Cat No. 60027-1-Ig
NQO1	Proteintech, Wuhan, China	Cat No. 67240-1-Ig
HO-1	Proteintech, Wuhan, China	Cat No. 10701-1-AP
GCLM	Proteintech, Wuhan, China	Cat No. 14241-1-AP
COL1A2	Proteintech, Wuhan, China	Cat No. 14695-1-AP
P21	Proteintech, Wuhan, China	Cat No. 10355-1-AP
γH2AX	Beyotime, Shanghai, China	AF5836

Table S4 Primer sequences were utilized in this study.

Primer name	Sequence
Nrf2	F: 5′-GCCGCTTAGAGGCTCATCTC-3′
	R: 5′-TGGGCGGCGACTTTATTCTT-3′
HO-1	F: 5′-CAAGCGCTATGTTCAGCGAC−3′
	R: 5′-GCTTGAACTTGGTGGCACTG−3′
NQO-1	F: 5′-GGCATCCTGCGTTTCTGTG-3′
	R: 5′-GGTTTCCAGACGTTTCTTCCAT-3′
GCLM	F: 5′-TTGGAGTTGCACAGCTGGATTC-3′
	R: 5′- TGGTTTTACCTGTGCCCACTG-3′
MMP-1	F: 5'-GATTCGGGGAGAAGTGATGTTC-3'
	R: 5'-CTCCTTTGGCTTCCCTAGAACT-3'
MMP-9	F: 5'-GCACCACCACAACATCACCTAT-3'
	R: 5'-GATGACGAGTTGTGGTCCCTG-3'
COL3A1	F: 5′-AGGACAAGAGGCATGTCTGGTT-3′
	R: 5′-TTGCAGTGGTAGGTGATGTTCTG-3′
IL-1β	F: 5′-TCCAGGATGAGGACATGAGCAC-3′
	R: 5′-GAACGTCACACACCAGCAGGTTA-3'
TNF-α	F: 5′-ACTCCAGGCGGTGCCTATGT-3′
	R: 5′-GTGAGGGTCTGGGCCATAGAA-3'
GAPDH	F: 5′-CAAGCGCTATGTTCAGCGAC−3′
	R: 5′-GCTTGAACTTGGTGGCACTG−3′
IL-6	F: 5′-ACTCACCTCTTCAGAACGAATTG-3′
	R: 5′-CCATCTTTGGAAGGTTCAGGTTG-3′
P21	F: 5′-CTGAGCCGCGACTGTGATGCG-3′
	R: 5′-GGTCTGCCGCCGTTTTCGACC-3′
P16	F: 5′-GCAGCATGGAGCCTTCGGCT-3′
	R: 5′-TGCAGCACCACCAGCGTGTC-3′
JAK2	F: 5′-CGAATGGTGTTTCTGATGTACC-3′
	R: 5′-GTCTCCTACTTCTCTTCGTACG-3′
SOCS1	F:5′-TCTCACCTCTTGAGGGGGTC-3′
	R: 5′-AGAGGTAGGAGGTGCGAGTT-3′
C-Myc	F: 5′-CATCAGCACAACTACGCAGC-3′
	R: 5′-GCTGGTGCATTTTCGGTTGT-3′

STAT1	F: 5′-TGTATGCCATCCTCGAGAGC-3′
	R: 5′-AGACATCCTGCCACCTTGTG-3′
STAT2	F: 5′-CCGGGACATTCAGCCCTTTT-3′
	R: 5′-CTCATGTTGCTGGCTCTCCA-3′
IRF9	F: 5′-TTCTTCAAGGCCTGGGCAAT-3′
	R: 5′-CCTGGTGGCAGCAACTGATA-3′

49
